# Analysis on the Differences of Combination Effects of Science and Technology Innovation Policies

**DOI:** 10.1155/2022/9011795

**Published:** 2022-03-30

**Authors:** Meirong Zhou, Ping Wei, Xi Zeng, Lianbing Deng

**Affiliations:** ^1^Zhuhai Da Hengqin Science and Technology Development Co., Ltd., Zhuhai 519031, China; ^2^School of Economics, Huazhong University of Science and Technology, Wuhan 430000, China

## Abstract

Science and technology innovation (STI) policy is a strategic principle to guide the whole cause of STI. The study on STI policy and its effect is particularly important. Most of the existing studies on the effect of STI policy focus on the effect of a single policy, and the studies on the effect of policy combination and its differences need to be further enriched and improved. This study proposes a method combining system simulation experiment and analysis of variance (ANOVA) to study the differences of combination effects of STI policies. The results show that there are significant effect differences in the combination of STI policies as a whole, but when it comes to different combinations of STI policies, not all policy combinations have significant differences. This study not only points out whether there are significant differences in a certain effect among which combinations of STI policies but also points out whether there are significant differences in all effects among which combinations of STI policies at the same time. This study has theoretical and practical significance for realizing scientific policy-making and sustainable development.

## 1. Introduction

STI is the inexhaustible driving force for the development of modern countries and the fundamental driving force for realizing sustainable economic development. At present, the improvement of STI ability and the high-quality development of STI have become the theme of the times. Nowadays, countries all over the world attach great importance to their own STI. Effective innovation and high-level patented technology have become important indicators to measure the level of national economic development and comprehensive national strength. In order to accelerate the pace of STI, countries all over the world have formulated STI policies to promote the development of innovation activities and the transformation of achievements. STI policy is the basic action criterion stipulated by a country to realize the STI task in a certain historical period. It is the strategic principle to determine the development direction of STI and guide the whole STI [1]. STI policy can ensure the effective implementation of various innovation activities and the rational allocation of innovation resources. Its reasonable formulation will help to improve innovation performance, so as to promote the construction of national innovation system. As a key factor to strengthen the national scientific and technological strategic force and drive the development of STI, STI policy has attracted extensive attention in the academic circles and has become an important research topic [2].

After studying the existing literature, it is found that the research on policy effects (including STI policy effects) related to this research is mainly reflected in the following two aspects:

1. What methods are used to study policy effects? The existing research methods on policy effects (including STI policy effects) are divided into two categories: qualitative research methods and quantitative research methods. Most of them analyze the impact of policies from a qualitative perspective. For example, Surianto et al. studied why policy efforts to reduce disaster risk often fail to improve future disaster relief [3]; Krivosheev et al. studied the effectiveness of EU policy in Walloon, Belgium, and the economic development of the region compared with Flanders, another Belgian region [4]. Based on the current situation of industrial transfer in Guangdong Province, Shi et al. discussed the role of policies in promoting industrial transfer and existing problems, and put forward suggestions on the innovation of regional policies in Guangdong Province [5]; Yu [6] and Lan et al. [7] studied the impact of policies on regional economic development and believed that the government should formulate regional economic policies in line with its own regional economic development direction according to the economic development of each region, so as to scientifically and reasonably allocate the resources of each region; Lin studied the impact of EU policies on Portugal's national STI capacity [8]. In addition, a few of them analyze the impact of policies from a quantitative perspective by establishing models. Wang et al. used system dynamics to build a mechanism model of the impact of STI policies on regional innovation capability and studied the impact mechanism of different policies on regional innovation capability [9]; Avdiushchenko and Zając proposed a set of possible indicators to evaluate the progress of EU countries in realizing circular economy at the regional level [10]; O'Brien and Burrows used vertical and mixed methods to analyze the effectiveness of regional policies for large-scale layoffs [11]; Gülal and Ayaita took the introduction of minimum wage in Germany as the standard experiment and used the double difference model to analyze the impact of minimum wage on well-being [12].
2. Research on the effect of single policy: most of the existing research on the effect of STI policy is to study the effect of a single STI policy. Cheng et al. found that government research and development (R&D) investment and human investment have a positive effect on STI, while enterprise financing policy has a significant negative effect on STI [13]; Deng and Long [14] and Yu [15] pointed out that R&D investment has a positive impact on STI; Fan [16] and Wang and Deng [17] believed that intellectual property protection helps promote STI; Zhuang et al. emphasized that the environmental policy of STI has a significant impact on STI [18]; Álvarez-Ayuso et al. studied the impact of tax credit on R&D [19]; Raffaello and Paolo studied the innovation effect of R&D subsidy policy in northern Italy [20]; Mukherjee et al. studied the relationship between taxation and innovation and pointed out that taxation hinders innovation [21]; Cappelen et al. believed that tax incentives have little effect on the marketization of new products and patented products, but they are conducive to the innovation of product production process and the formation of new products [22]; Guerzoni and Raiteri took enterprises in EU Member States as samples, and considered that government procurement not only stimulates enterprises' R&D investment, but also stimulates innovation output [23]; Aschhoff and Sofka empirically tested the effect of government procurement on innovation output by using the survey data of German enterprises and the results show that government procurement can promote enterprise innovation [24]; Song and Zhang studied the relationship between government procurement policies and implementation rules to promote independent innovation, combed the practices in typical countries, and pointed out that government procurement is an important policy tool to support the innovation [25]; Hu et al. used provincial panel data to conduct an empirical test on whether China's government procurement has produced the expected technological innovation effect and the results show that China's government procurement not only does not promote innovation, but hinders innovation [26]. At present, there are also a few studies on the combination effect of STI policies. Kalcheva et al. used the triple difference method to study the impact of the combination of supply policy and demand policy on innovation [27]; Dou et al. found that the combination of STI policies has a significant impact on enterprise technological innovation [28]; Guo et al. believed that the combination effect of various STI policies is better than a single type of policies. When the policies are issued, we should pay attention to the complementarity and systematicness of the policies and avoid focusing too much on a certain link [29].

To sum up, the existing research on the effect of STI policy is mainly the effect of a single policy, and the research on the combination effect of STI policy needs to be further enriched and improved. In fact, the effect of STI policy is often the result of the joint force of multiple policies. At the same time, the existing research on the combination effect of STI policy needs to pay more attention to the research on the difference of policy combination effect. The detailed analysis of the difference of policy combination effect is helpful for researchers and policy makers to have a deeper understanding of the effect of policy combination, and it is also helpful for scientific policy implementation. In view of this, this study will use the ANOVA method to study the differences of the combination effects of STI policies.

The above is the first part of this study, that is, the introduction. This study will be carried out according to the following structure: firstly, the research methods used are introduced, that is, the one-way ANOVA method and multiple comparison method are introduced; secondly, the differences of policy portfolio effects are analyzed in detail by using one-way ANOVA and multiple comparison method; the last part is the summary of this study.

## 2. Relevant Theoretical Basis

Because STI has the characteristics of externality and risk of public goods, it will hinder the enthusiasm of the main body of STI, and lead to the fact that the supply quantity in the market is often difficult to reach the social optimal level. This provides a theoretical basis for the government to stimulate the innovation power of STI subjects through policies [30]. Market failure theory holds that in the case of market failure, it is difficult to achieve Pareto optimal state only by market domination. At this time, it is necessary to combine the market with the government. R&D and innovation activities have the characteristics of externality and risk of public goods to a certain extent, which will lead to the failure of STI market, and provide a theoretical basis for the government to intervene in the R&D activities of STI subjects. Endogenous growth theory is based on Schumpeter's innovation theory, takes technological progress as an endogenous variable, and believes that human capital and knowledge accumulation bring the increasing marginal rate of return, so as to ensure economic growth and social progress. These endogenous factors that bring about the economic growth will be affected by government policies and are sensitive to policies. Romer [31] pointed out that we should vigorously promote various policies to promote innovation, such as giving direct subsidies to knowledge output, paying attention to education, strengthening intellectual property protection, etc. Through these policy orientations, we should send signals to STI subjects and stimulate their R&D enthusiasm.

## 3. Research Method: One-Way ANOVA and Multiple Comparison

ANOVA is used to test the significance of the difference between the mean of two or more samples. Its basic idea is as follows: by analyzing and decomposing the fluctuation of experimental data, and then comparing the possible systematic fluctuation and random fluctuation between each group of experimental data under a certain influencing factor, it is inferred whether there is a significant difference between the overall mean values. If there is a significant difference, it indicates that the influence of this factor is significant; otherwise, it is not significant [32, 33]. When the influence of only one factor on the experimental results is considered, it is called one-way ANOVA; when two factors affect the experimental results, it is called two-way ANOVA; by analogy, when more than two factors affect the experimental results, it is called multiway ANOVA.

The multiple comparison method in the parameter method is to test which overall mean values are equal and which overall mean values are different through pairwise comparison between the overall mean values. There are many methods for multiple comparison. In this study, the least significant difference (LSD) method proposed by Fisher is used. It is a simple deformation of *t*-test. It makes full use of sample information in the calculation of standard error and uniformly estimates a more robust standard error for the mean of all groups [34].

### 3.1. Related Concepts and Basic Assumptions of One-Way ANOVA

The one-way ANOVA method involves three terms: factor, level, and observation. Their meanings are as follows:

1. Factor, also known as condition, refers to the object to be tested in the ANOVA
2. Level, also known as treatment, refers to different values corresponding to factors
3. Observation, refers to the experimental data obtained at each level of the factor

The one-way ANOVA method has the following basic assumptions:

1. The assumption of normality is that each population obeys the normal distribution. For each level of factors, the observed values are simple random samples from the normal population.
2. The homogeneity of variance is assumed, that is, the variance of each population should be equal. For each group of observation data, they are extracted from the normal distribution of the same variance.
3. Independence assumption, that is, the observations are independent of each other.

### 3.2. Basic Process of One-Way ANOVA

**Figure 1:**
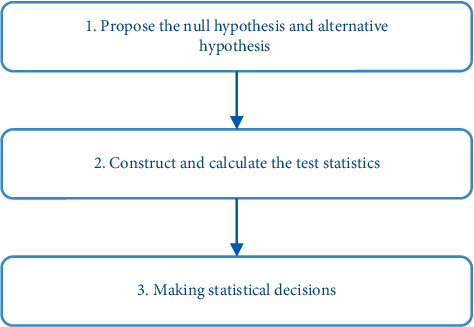
Basic process of one-way ANOVA.

When the one-way ANOVA method is used for research analysis, the basic process shown in Figure 1 can be adopted.

Firstly, the null hypothesis and alternative hypothesis are put forward. If the factor to be tested has *k* levels, and the corresponding mean value of each level is *u*_*i*_, where *i* = 1, 2,…, *k*, then the null hypothesis and alternative hypothesis are as follows:

*H*_0_: *u*_1_ *=* *u*_2_ *=* … *=* *u*_*k*_ (independent variables have no significant impact on dependent variables);

*H*_1_: *u*_1_, *u*_2_,…, *u*_*k*_ are not all equal (independent variables have a significant impact on dependent variables).

The null hypothesis (*H*_0_) means that the factor to be tested has no impact on the experimental results, while the alternative hypothesis (*H*_1_) means that the factor to be tested has an impact on the experimental results.

Secondly, the test statistics are constructed and calculated. In order to test whether the null hypothesis (*H*_0_) is true, it is necessary to construct appropriate test statistics first. Specifically, it is necessary to construct three sums of squares of error, which are sum of squares of total error (SST), sum of squares of factor error (SSA) and sum of squares of random error (SSE). Let *x*_*ij*_ represent the *j*-th observation value of the *i*-th level, then the calculation formulas of these three sums of squares of error are

(1) $$\begin{array}{c} \text{SST}=\overset{k}{\underset{i=1}{\sum }}\overset{n_{i}}{\underset{j=1}{\sum }}{\left(x_{ij}-\overset{=}{x}\right)}^{2},\\ \text{SSA}=\overset{k}{\underset{i=1}{\sum }}\overset{n_{i}}{\underset{j=1}{\sum }}{\left({\bar{x}}_{i}-\overset{=}{x}\right)}^{2}=\overset{k}{\underset{i=1}{\sum }}n_{i}{\left({\bar{x}}_{i}-\overset{=}{x}\right)}^{2},\\ \text{SSE}=\overset{k}{\underset{i=1}{\sum }}\overset{n_{i}}{\underset{j=1}{\sum }}{\left(x_{ij}-{\bar{x}}_{i}\right)}^{2},\\ {\bar{x}}_{i}=\frac{\sum_{j=1}^{n_{i}}x_{ij}}{n_{i}},\\ \overset{=}{x}=\frac{\sum_{i=1}^{k}\sum_{j=1}^{n_{i}}x_{ij}}{n}=\frac{\sum_{i=1}^{k}n_{i}{\bar{x}}_{i}}{n}, \end{array}$$

where ${\bar{x}}_{i}$ represents the mean value of the factor at Level *i* and *n*_*i*_ is the number of the *i*-th overall experimental data, *i* = 1, 2,…, *k*; $\bar{x}$ represents the mean value of all observations, *n* *=* *n*_1_*+n*_2_*+*⋯+*n*_*k*_.

These three sums of squares of error satisfy the following identity relationship.

(2) $$\begin{array}{c} \mathrm{SST=SSA+SSE}. \end{array}$$

The sum of squares of error divided by their corresponding degrees of freedom is called mean square error. The degree of freedom of *SST* is *n* − 1, where *n* is the number of all observed values. The degree of freedom of *SSA* is *k* − 1, and its corresponding mean square error is usually recorded as MSA, where *k* is the number of factor levels. The degree of freedom of SSE is *n* − *k*, and its corresponding mean square error is usually recorded as MSE. Thus, the value of *F* = MSA/MSE can be obtained, and it obeys the *F* (*k* − 1, *n* − *k*) distribution.

Finally, make statistical decisions. According to the given significance level *α*, check the *F* distribution table to determine *F*_*α*_ (*k* − 1, *n* − *k*). If *F* > *F*_*α*_ (or *P* < *α*), then reject the null hypothesis, indicating that the factor has a significant impact on the experimental results. Otherwise, accept the null hypothesis and consider that the factor has no significant impact on the experimental results.

### 3.3. Basic Flow of LSD Method

When the one-way ANOVA method is used for research analysis, the basic process shown in Figure 1 can be adopted.

After one-way ANOVA, if you want to know which population means are equal and which population means are different, you need to make multiple comparisons. In this study, LSD method is used for multiple comparison, and its basic steps are similar to one-way ANOVA, as follows:

Firstly, the null hypothesis and alternative hypothesis are put forward.

*H*_0_: *u*_*i*_ = *u*_*j*_;

*H*_1_: *u*_*i*_, *u*_*j*_ are not equal where 1 ≤ *i* < *j* ≤ *k*.

Secondly, the test statistics are constructed and calculated. The test statistic of LSD method is *t* statistic, and its calculation formula is

(3) $$\begin{array}{c} t=\frac{\left({\bar{x}}_{i}-{\bar{x}}_{j}\right)-\left(u_{i}-u_{j}\right)}{\sqrt{MSE\left(1/n_{i}+1/n_{j}\right)}}. \end{array}$$

*t* statistic obeys the *t* distribution with *n* − *k* degrees of freedom.

Finally, make statistical decisions. According to the given significance level *α*, check the *t* distribution table to determine *t*_*α/2*_ (*n* − *k*). If |*t*| > *t*_*α/2*_(*n* − *k*) (or *P* < *α*), then reject the null hypothesis, indicating that there is a difference between the two population mean values. Otherwise, accept the null hypothesis and consider that there is no difference between the two population mean values.

### 3.4. Welch's ANOVA and Games–Howell Test

It is worth noting that in this study, when the variances are unequal, we will use Welch's ANOVA method to replace the aforementioned one-way ANOVA method for corresponding analysis, because Welch statistic is better than *F* statistic when the variance is unequal [35]. At the same time, we will use the Games–Howell test method to replace the above LSD method for corresponding analysis. For a detailed introduction to the Games–Howell test method, please refer to reference [36].

## 4. Analysis on the Differences of Combination Effects

This study is based on our previous research achievement “Analysis on the combination effect of science and technology innovation policy: from the perspective of system simulation” (hereinafter referred to as Achievement A), which proposes a system simulation method to analyze the combination effect of STI policy. This study uses the system simulation model in Achievement A to carry out simulation experiments and obtain simulation experimental data, so as to analyze the differences of policy combination effects. The following is a brief introduction to the system simulation model in Achievement A.

**Figure 2:**
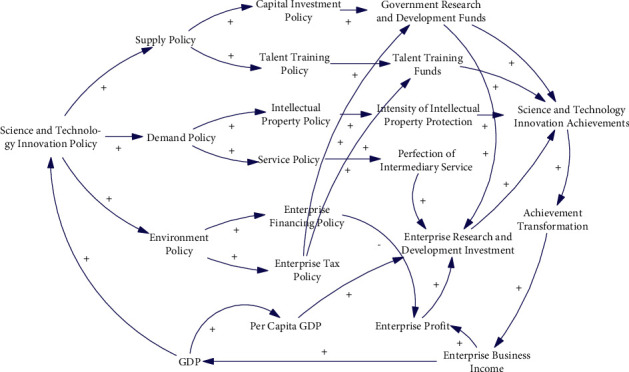
Conceptual model.

**Figure 3:**
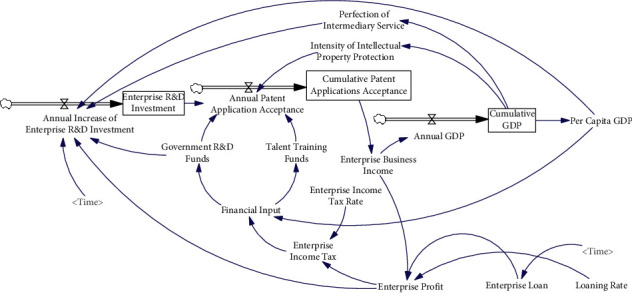
Simulation model.

Rothwell and Zegveld [37] believe that the policy effect originates from different levels. Therefore, the STI policy can be divided into three categories, one is supply policy, and the other two are demand policy and environmental policy. In this study, our goal is to analyze the impact of different level STI policies. Therefore, this classification method will be adopted. Considering the quantification of policies and the availability of data, the STI supply policy in Achievement A only considers the capital investment policy and talent training policy, the STI demand policy only considers the intellectual property policy and STI service policy, and the STI environment policy only considers the enterprise financing policy and enterprise tax policy. The Achievement A establishes the conceptual model as shown in Figure 2, that is, the causality diagram. The system simulation model corresponding to the conceptual model, that is, the stock flow diagram, is shown in Figure 3.

The above is a brief introduction to the system simulation model in Achievement A. For the detailed introduction of Achievement A, please refer to my previous research Achievement A.

It is worth noting that Achievement A has conducted an empirical study using the relevant data of STI in Guangdong Province from 2010 to 2019. Its system simulation model does not consider the uncertain factors, but this study will take the uncertain factors into account. Specifically, this study will add random distribution to the expression of relevant variables in Achievement A, as shown in Table 1. For example, for the annual GDP, the expression in this study is: annual GDP = the expression of annual GDP in Achievement A + RANDOM NORMAL (−5460.12, 5291.63, 0, 3622.37). Among them, RANDOM NORMAL(min, max, mean, stdev) is the normal distribution form in the system dynamics simulation software VENSIM, which needs to input four parameters.

It can be seen from Table 1 that all the random distributions in the expressions of Variable 1 to 9 are a normal distribution, because the expressions corresponding to these variables in Achievement A are obtained by linear regression analysis method, which requires that the error term obey a normal distribution and the mean value is 0. In Achievement A, the expressions of Variable 10 to 13 are obtained by weighted regression analysis. This study assumes that the error terms of variables 10 to 13 obey a normal distribution. The parameters of the random distribution shown in Table 1 are obtained by analyzing the error term.

This study will make a difference analysis on the combination effects of the above three types of policies in SPSS statistical analysis software. The effects here mainly refer to the economic effect and STI effect. The per capita GDP and patent application acceptance are used as the effect indicators respectively. In this study, each type of policy takes three levels. Specifically, the three levels of supply policy are benchmark level, relative benchmark increase of 5% and relative benchmark decrease of 5%. The three levels of demand policy are similar to those of supply policy. The three levels of environmental policy are the benchmark level, the relative benchmark increase of 1% and the relative benchmark decrease of 1%. The benchmark levels for each policy are detailed in Achievement A. Since there are three types of policies, and each type of policy has three levels, this study will design 3^3^ = 27 simulation experiments, as shown in Table 2.

### 4.1. Descriptive Statistics

After the preliminary analysis of the data (that is, the model output data with the simulation time of 2025) obtained from the above simulation experiments, some descriptive statistical analysis results are obtained as shown in Table 3. It is not difficult to find from Table 3 that the number of experiments in each experiment is the same, 50 times. From Table 3, we can also see the mean value and standard deviation of per capita GDP (PCGDP) and annual patent application acceptance (APAA) of each simulation experiment.

### 4.2. Test of Normality and Test of Homogeneity of Variance

Because the one-way ANOVA method requires that the population should meet the normality and homogeneity of variance, it is necessary to test the normality and homogeneity of variance before analyzing the differences of policy combination effects. It can be seen from Table 1 that the normality requirements are met. From Table 4, it can be seen that the significance (*P* value) of per capita GDP is greater than 0.05. Thus, the homogeneity of variance is met at this time. For the annual patent application acceptance, the significance (*P* value) is less than 0.05. Hence, the homogeneity of variance is not met at this time.

### 4.3. Difference Analysis of Economic Effect

For the per capita GDP, since each population meets the requirements of normality and homogeneity of variance, we use the abovementioned one-way ANOVA method for analysis. As can be seen from Table 5, the significance (*P* value) is less than 0.05, which indicates that there are significant differences in the economic effect (i.e., per capita GDP) of various combinations of STI policies. It is worth noting that this significant difference refers to the significant difference of the whole. At this time, we do not know which combinations have significant differences, and which combinations have no significant differences. In order to answer this question, it is necessary to make multiple comparisons so that we can clearly understand which combinations have significant differences. It can be seen from Table 6 that when 0.05 is taken as the significance level, there is a significant difference between the economic effect of the policy combination corresponding to Experiment 1 and the economic effect of other policy combinations, except that it is not significantly different from the economic effect of the policy combination corresponding to Experiment 7, 10, 13, 19, and 25. In addition to the policy combination corresponding to Experiment 1, similar difference analysis can also be carried out for other policy combinations in combination with Table 6, which will not be repeated here.

### 4.4. Difference Analysis of STI Effect

For the annual patent application acceptance, although each population meets the requirements of normality, they do not meet the requirements of homogeneity of variance. Therefore, the abovementioned one-way ANOVA method is not applicable here. At this time, we can use the abovementioned Welch's ANOVA method for analysis. As can be seen from Table 7, the significance (*P* value) is less than 0.05, which shows that the STI effect (i.e., annual patent application acceptance) of various combinations of STI policies are significantly different as a whole. In order to further understand which population mean values are equal and which population mean values are different, it is necessary to make multiple comparisons using the aforementioned Games–Howell test method. It is not difficult to find from Table 8 that, at the significance level of 0.05, there is no significant difference between the STI effect of the policy combination corresponding to Experiment 1 and the STI effect of the policy combination corresponding to Experiment 4, 7, 10, 13, 16, 19, 22, and 25. However, there are significant differences with other policy combinations in the effect of STI. In addition to the policy combination corresponding to Experiment 1, similar difference analysis can also be carried out for other policy combinations in combination with Table 8, which will not be repeated here.

### 4.5. Comprehensive Analysis of Differences

The above analyzes the differences of the economic effect and STI effect of each combination of STI policies. Next, we take policy Combination 1 as an example and analyze these two effects of each combination of STI policies at the same time. By sorting out Tables 6 and 8, we can get Table 9. It can be seen from Table 9 that the economic effect and STI effect of policy Combination 1 are significantly different from those of policy Combination 2, 3, 5, 6, 8, 9, 11, 12, 14, 15, 17, 18, 20, 21, 23, 24, 26 and 27, but not from those of policy Combination 7, 10, 13, 19 and 25. In addition, there are significant differences in economic effect between policy Combination 1 and policy Combination 4, 16 and 22, but there is no significant difference in STI effect.

## 5. Conclusions

### 5.1. Research Summary

STI has become an important driving force for national and regional economic and social development. As a tool for the government to guide and promote the promotion of scientific and technological competitiveness, STI policy has a significant impact on STI. Most of the existing studies focus on the effect of a single policy, and the studies on the effect of policy combination and its differences need to be further enriched and improved. Under this background, this study proposes a method combining system simulation experiment and ANOVA to study the differences of the combination effects of STI policies. The research is summarized as follows:

1. On the whole, there are significant differences in both the economic effect of STI policy combination and the STI effect of STI policy combination.
2. Specific to different combinations of STI policies, not all combinations of STI policies have significant differences in policy effects.
3. There are significant differences in the economic effect of some STI policy combinations, but the differences in the STI effect are not significant. There are significant differences in STI effect among some STI policy combinations, but the differences in economic effect are not significant.
4. This study not only points out whether there are significant differences in economic effect or STI effect among STI policy combinations, but also points out whether there are significant differences in economic effect and STI effect among STI policy combinations at the same time.

### 5.2. Policy Implications

This study gives us the following enlightenment:

Firstly, the empirical results show that there are significant differences in policy effects among some STI policy combinations, while there are no significant differences in policy effects among some STI policy combinations. Therefore, policymakers should fully consider the differences in policy effects among policy combinations when formulating policies, so as to avoid making useless efforts in formulating policies. In other words, policymakers should avoid formulating policies that have no significant difference in policy effects from the original policies.

Secondly, the empirical results show that the economic effect and STI effect of STI policy combinations are not always synchronous, that is, sometimes there are significant differences in one effect but not in the other. Therefore, when making policies, policy makers sometimes have to make a trade-off among a variety of policy effects.

### 5.3. Contribution and Prospect

The contributions of this study are as follows:

1. The existing research mainly focuses on the effect of a single policy, and the research on the combination effect of STI policy, especially the research on the difference of policy combination effect, needs to be further enriched and improved. This research enriches and expands the relevant literature research, makes up for the shortcomings of previous research, and provides an effective supplement for the existing research on STI policy.
2. This study proposes a method combining system simulation experiment and ANOVA to study the differences of the combination effects of STI policies. This method based on the combination of system simulation and data analysis and mining enhances the modeling and solving ability of practical problems, provides a reference framework for similar research, and reflects the characteristics of this study in research methods.

This study provides enlightenment for policy makers to fully understand the effect of STI policy and make appropriate adjustment. At the same time, it enriches and expands the relevant literature research, which has certain theoretical and practical significance. However, this study also has some limitations. For example, only six kinds of STI policies are considered, and other STI policies are not deeply studied. In future research, more STI policies can be included in the research.

## Figures and Tables

**Table 1:** Random distribution in variable expressions.

Number	Variable name	Random distribution to be added
Variable 1	Annual GDP	RANDOM NORMAL (−5460.12, 5291.63, 0, 3622.37)
Variable 2	Enterprise loan	RANDOM NORMAL (−6092.92, 8022.4, 0, 5002.37)
Variable 3	Enterprise income tax	RANDOM NORMAL (−107.338, 67.0867, 0, 52.0629)
Variable 4	Perfection of intermediary service	RANDOM NORMAL (−1.2481, 0.755273, 0, 0.789901)
Variable 5	Enterprise business income	RANDOM NORMAL (−12078.2, 13275.6, 0, 8515.95)
Variable 6	Talent training funds	RANDOM NORMAL (−213.521, 161.98, 0, 126.222)
Variable 7	Government R&D funds	RANDOM NORMAL (−118.463, 103.843, 0, 76.0717)
Variable 8	Intensity of intellectual property protection	RANDOM NORMAL (−0.184447, 0.11458, 0, 0.117456)
Variable 9	Per capita GDP	RANDOM NORMAL (−1081.07, 1585.02, 0, 838.532)
Variable 10	Annual increase of enterprise R&D investment	RANDOM NORMAL (−71.0683, 19.1128, −8.75484, 25.325)
Variable 11	Annual patent application acceptance	RANDOM NORMAL (−129351, 50584.4, −6503, 46332)
Variable 12	Enterprise profit	RANDOM NORMAL (−362.065, 1172.9, 77.7868, 427.839)
Variable 13	Financial input	RANDOM NORMAL (−661.539, 1540.54, 66.5193, 658.025)

**Table 2:** Simulation experiment.

Experiment	Supply policy	Demand policy	Environmental policy
Experiment 1	Benchmark	Benchmark	Benchmark
Experiment 2	Increase of 5%	Benchmark	Benchmark
Experiment 3	Decrease of 5%	Benchmark	Benchmark
Experiment 4	Benchmark	Increase of 5%	Benchmark
Experiment 5	Increase of 5%	Increase of 5%	Benchmark
Experiment 6	Decrease of 5%	Increase of 5%	Benchmark
Experiment 7	Benchmark	Decrease of 5%	Benchmark
Experiment 8	Increase of 5%	Decrease of 5%	Benchmark
Experiment 9	Decrease of 5%	Decrease of 5%	Benchmark
Experiment 10	Benchmark	Benchmark	Increase of 1%
Experiment 11	Increase of 5%	Benchmark	Increase of 1%
Experiment 12	Decrease of 5%	Benchmark	Increase of 1%
Experiment 13	Benchmark	Increase of 5%	Increase of 1%
Experiment 14	Increase of 5%	Increase of 5%	Increase of 1%
Experiment 15	Decrease of 5%	Increase of 5%	Increase of 1%
Experiment 16	Benchmark	Decrease of 5%	Increase of 1%
Experiment 17	Increase of 5%	Decrease of 5%	Increase of 1%
Experiment 18	Decrease of 5%	Decrease of 5%	Increase of 1%
Experiment 19	Benchmark	Benchmark	Decrease of 1%
Experiment 20	Increase of 5%	Benchmark	Decrease of 1%
Experiment 21	Decrease of 5%	Benchmark	Decrease of 1%
Experiment 22	Benchmark	Increase of 5%	Decrease of 1%
Experiment 23	Increase of 5%	Increase of 5%	Decrease of 1%
Experiment 24	Decrease of 5%	Increase of 5%	Decrease of 1%
Experiment 25	Benchmark	Decrease of 5%	Decrease of 1%
Experiment 26	Increase of 5%	Decrease of 5%	Decrease of 1%
Experiment 27	Decrease of 5%	Decrease of 5%	Decrease of 1%

**Table 3:** Descriptive statistical analysis of effect indicators.

		*N*	Mean	Std. dev	95% CI for mean		Min	Max
					LB	UB		
PCGDP	1	50	116875.19	2756.01	116091.94	117658.44	110202.14	122390.17
	2	50	119737.81	3000.28	118885.14	120590.48	112699.75	126021.47
	3	50	114350.49	2552.16	113625.18	115075.81	107994.00	119207.41
	4	50	117970.75	2781.64	117180.22	118761.28	111239.44	123558.27
	5	50	120891.43	3030.46	120030.19	121752.68	113788.03	127260.36
	6	50	115395.78	2574.09	114664.23	116127.32	108986.92	120314.95
	7	50	115786.38	2730.63	115010.35	116562.42	109171.17	121229.48
	8	50	118591.91	2970.42	117747.72	119436.09	111618.66	124791.30
	9	50	113311.15	2530.45	112592.00	114030.30	107006.61	118106.33
	10	50	116676.04	2769.68	115888.91	117463.17	109978.14	122227.02
	11	50	119551.25	3019.44	118693.13	120409.36	112478.59	125886.61
	12	50	114144.73	2561.82	113416.67	114872.79	107770.75	119026.17
	13	50	117778.65	2796.14	116983.99	118573.30	111020.88	123404.47
	14	50	120713.78	3050.69	119846.79	121580.78	113573.70	127137.38
	15	50	115195.57	2584.37	114461.10	115930.04	108768.08	120140.98
	16	50	115580.43	2743.49	114800.74	116360.12	108941.84	121057.23
	17	50	118396.74	2988.53	117547.41	119246.07	111390.78	124644.86
	18	50	113100.02	2539.49	112378.31	113821.73	106779.22	117917.95
	19	50	117037.61	2740.57	116258.75	117816.47	110393.23	122511.11
	20	50	119880.62	2978.97	119034.00	120727.23	112881.50	126106.11
	21	50	114525.60	2541.09	113803.43	115247.77	108189.75	119353.38
	22	50	118125.65	2765.38	117339.73	118911.56	111424.56	123669.33
	23	50	121024.80	3008.07	120169.91	121879.68	113962.31	127332.38
	24	50	115564.91	2562.37	114836.69	116293.13	109177.98	120453.05
	25	50	115956.06	2715.99	115184.18	116727.93	109367.95	121359.86
	26	50	118743.84	2950.15	117905.41	119582.26	111807.53	124887.91
	27	50	113492.03	2519.99	112775.86	114208.20	107206.84	118259.78
	Total	1350	116977.75	3635.29	116783.65	117171.84	106779.22	127332.38

APAA	1	50	5447216.71	566848.37	5286120.19	5608313.23	4123422.50	6897111.50
	2	50	6781777.86	726435.97	6575327.04	6988228.68	5128423.50	8586776.00
	3	50	4411721.91	449265.41	4284042.09	4539401.73	3340311.75	5586297.50
	4	50	5727187.33	585571.63	5560769.71	5893604.95	4358983.50	7220039.00
	5	50	7124829.99	751794.28	6911172.42	7338487.56	5413846.50	8985734.00
	6	50	4643638.21	463370.31	4511949.82	4775326.59	3537511.25	5851589.00
	7	50	5174886.30	548610.50	5018972.91	5330799.68	3894416.50	6582780.00
	8	50	6448667.84	701800.64	6249218.30	6648117.38	4851419.00	8199133.00
	9	50	4185764.68	435493.88	4061998.69	4309530.67	3148286.50	5327639.00
	10	50	5452400.10	577314.29	5288329.19	5616471.01	4106576.00	6928417.00
	11	50	6814753.65	743851.16	6603353.49	7026153.81	5126440.50	8659248.00
	12	50	4400640.36	455516.10	4271184.11	4530096.60	3315451.00	5592566.00
	13	50	5739246.96	597028.50	5569573.34	5908920.58	4346903.50	7260298.50
	14	50	7167935.57	770751.97	6948890.28	7386980.86	5418855.50	9071408.00
	15	50	4637260.98	470270.14	4503611.68	4770910.28	3515921.25	5864043.00
	16	50	5173637.43	558134.43	5015017.38	5332257.49	3873134.75	6605654.50
	17	50	6472158.41	717754.95	6268174.71	6676142.11	4842897.00	8259190.00
	18	50	4170287.47	441123.80	4044921.47	4295653.47	3120412.50	5328120.50
	19	50	5428011.83	555576.99	5270118.60	5585905.06	4128479.50	6850074.50
	20	50	6730683.09	708049.78	6529457.57	6931908.61	5115007.50	8494183.00
	21	50	4412000.78	442373.58	4286279.60	4537721.96	3356138.50	5567822.00
	22	50	5700801.13	573329.26	5537862.76	5863739.50	4358943.00	7163762.50
	23	50	7063271.32	731911.68	6855264.32	7271278.32	5393022.50	8879698.00
	24	50	4638950.83	455836.91	4509403.41	4768498.24	3549818.75	5826734.00
	25	50	5162424.61	538262.52	5009452.09	5315397.13	3904222.50	6544449.00
	26	50	6407408.94	684837.86	6212780.17	6602037.71	4844911.50	8119192.50
	27	50	4190705.05	429214.88	4068723.53	4312686.57	3167381.50	5315209.50
	Total	1350	5544750.72	1157255.99	5482963.24	5606538.19	3120412.50	9071408.00

**Table 4:** Test of homogeneity of variances.

		Levene statistic	df1	df2	Sig.
PCGDP	Based on mean	0.479	26	1323	0.988
	Based on median	0.467	26	1323	0.990
	Based on median and with adjusted df	0.467	26	1302.907	0.990
	Based on trimmed mean	0.475	26	1323	0.989

APAA	Based on mean	3.182	26	1323	0.000
	Based on median	3.129	26	1323	0.000
	Based on median and with adjusted df	3.129	26	1116.829	0.000
	Based on trimmed mean	3.189	26	1323	0.000

**Table 5:** ANOVA.

Per_Capita_GDP					
	Sum of squares	df	Mean square	*F*	Sig.
Between groups	7638617410.110	26	293792977.312	38.148	0.000
Within groups	10188907629.601	1323	7701366.311		
Total	17827525039.710	1349			

**Table 6:** Multiple comparisons on dependent variable Per_Capita_GDP.

(*I*) experiment	(*J*) experiment	Mean difference (*I* − *J*)	Std. Error	Sig.	95% confidence interval	
					Lower bound	Upper bound
1	2	−2862.62^*∗*^	555.03	0.000	−3951.45	−1773.79
	3	2524.70^*∗*^	555.03	0.000	1435.87	3613.53
	4	−1095.56^*∗*^	555.03	0.049	−2184.39	−6.73
	5	−4016.24^*∗*^	555.03	0.000	−5105.07	−2927.41
	6	1479.42^*∗*^	555.03	0.008	390.59	2568.25
	7	1088.81	555.03	0.050	−0.02	2177.64
	8	−1716.71^*∗*^	555.03	0.002	−2805.54	−627.89
	9	3564.04^*∗*^	555.03	0.000	2475.22	4652.87
	10	199.15	555.03	0.720	−889.68	1287.98
	11	−2676.05^*∗*^	555.03	0.000	−3764.88	−1587.22
	12	2730.46^*∗*^	555.03	0.000	1641.63	3819.29
	13	−903.45	555.03	0.104	−1992.28	185.37
	14	−3838.59^*∗*^	555.03	0.000	−4927.42	−2749.76
	15	1679.62^*∗*^	555.03	0.003	590.79	2768.45
	16	1294.76^*∗*^	555.03	0.020	205.93	2383.59
	17	−1521.55^*∗*^	555.03	0.006	−2610.37	−432.72
	18	3775.17^*∗*^	555.03	0.000	2686.34	4864.00
	19	−162.42	555.03	0.770	−1251.25	926.41
	20	−3005.42^*∗*^	555.03	0.000	−4094.25	−1916.59
	21	2349.59^*∗*^	555.03	0.000	1260.76	3438.42
	22	−1250.45^*∗*^	555.03	0.024	−2339.28	−161.63
	23	−4149.60^*∗*^	555.03	0.000	−5238.43	−3060.77
	24	1310.29^*∗*^	555.03	0.018	221.46	2399.11
	25	919.13	555.03	0.098	−169.69	2007.96
	26	−1868.64^*∗*^	555.03	0.001	−2957.47	−779.81
	27	3383.16^*∗*^	555.03	0.000	2294.33	4471.99

2	1	2862.62^*∗*^	555.03	0.000	1773.79	3951.45
	3	5387.32^*∗*^	555.03	0.000	4298.49	6476.15
	4	1767.06^*∗*^	555.03	0.001	678.23	2855.89
	5	−1153.62^*∗*^	555.03	0.038	−2242.45	−64.79
	6	4342.03^*∗*^	555.03	0.000	3253.21	5430.86
	7	3951.42^*∗*^	555.03	0.000	2862.60	5040.25
	8	1145.90^*∗*^	555.03	0.039	57.07	2234.73
	9	6426.66^*∗*^	555.03	0.000	5337.83	7515.49
	10	3061.77^*∗*^	555.03	0.000	1972.94	4150.60
	11	186.56	555.03	0.737	−902.26	1275.39
	12	5593.08^*∗*^	555.03	0.000	4504.25	6681.91
	13	1959.16^*∗*^	555.03	0.000	870.33	3047.99
	14	−975.97	555.03	0.079	−2064.80	112.86
	15	4542.24^*∗*^	555.03	0.000	3453.41	5631.07
	16	4157.38^*∗*^	555.03	0.000	3068.55	5246.20
	17	1341.07^*∗*^	555.03	0.016	252.24	2429.90
	18	6637.79^*∗*^	555.03	0.000	5548.96	7726.62
	19	2700.20^*∗*^	555.03	0.000	1611.37	3789.03
	20	−142.81	555.03	0.797	−1231.63	946.02
	21	5212.21^*∗*^	555.03	0.000	4123.38	6301.04
	22	1612.16^*∗*^	555.03	0.004	523.33	2700.99
	23	−1286.99^*∗*^	555.03	0.021	−2375.81	−198.16
	24	4172.90^*∗*^	555.03	0.000	3084.07	5261.73
	25	3781.75^*∗*^	555.03	0.000	2692.92	4870.58
	26	993.97	555.03	0.074	−94.86	2082.80
	27	6245.78^*∗*^	555.03	0.000	5156.95	7334.61

3	1	−2524.70^*∗*^	555.03	0.000	−3613.53	−1435.87
	2	−5387.32^*∗*^	555.03	0.000	−6476.15	−4298.49
	4	−3620.26^*∗*^	555.03	0.000	−4709.09	−2531.43
	5	−6540.94^*∗*^	555.03	0.000	−7629.77	−5452.11
	6	−1045.28	555.03	0.060	−2134.11	43.55
	7	−1435.89^*∗*^	555.03	0.010	−2524.72	−347.06
	8	−4241.41^*∗*^	555.03	0.000	−5330.24	−3152.59
	9	1039.34	555.03	0.061	−49.48	2128.17
	10	−2325.55^*∗*^	555.03	0.000	−3414.38	−1236.72
	11	−5200.75^*∗*^	555.03	0.000	−6289.58	−4111.92
	12	205.76	555.03	0.711	−883.07	1294.59
	13	−3428.15^*∗*^	555.03	0.000	−4516.98	−2339.33
	14	−6363.29^*∗*^	555.03	0.000	−7452.12	−5274.46
	15	−845.08	555.03	0.128	−1933.91	243.75
	16	−1229.94^*∗*^	555.03	0.027	−2318.77	−141.11
	17	−4046.25^*∗*^	555.03	0.000	−5135.07	−2957.42
	18	1250.47^*∗*^	555.03	0.024	161.64	2339.30
	19	−2687.12^*∗*^	555.03	0.000	−3775.95	−1598.29
	20	−5530.12^*∗*^	555.03	0.000	−6618.95	−4441.29
	21	−175.11	555.03	0.752	−1263.94	913.72
	22	−3775.15^*∗*^	555.03	0.000	−4863.98	−2686.33
	23	−6674.30^*∗*^	555.03	0.000	−7763.13	−5585.47
	24	−1214.41^*∗*^	555.03	0.029	−2303.24	−125.59
	25	−1605.57^*∗*^	555.03	0.004	−2694.39	−516.74
	26	−4393.34^*∗*^	555.03	0.000	−5482.17	−3304.51
	27	858.46	555.03	0.122	−230.37	1947.29

4	1	1095.56^*∗*^	555.03	0.049	6.73	2184.39
	2	−1767.06^*∗*^	555.03	0.001	−2855.89	−678.23
	3	3620.26^*∗*^	555.03	0.000	2531.43	4709.09
	5	−2920.68^*∗*^	555.03	0.000	−4009.51	−1831.85
	6	2574.97^*∗*^	555.03	0.000	1486.15	3663.80
	7	2184.37^*∗*^	555.03	0.000	1095.54	3273.19
	8	−621.16	555.03	0.263	−1709.98	467.67
	9	4659.60^*∗*^	555.03	0.000	3570.77	5748.43
	10	1294.71^*∗*^	555.03	0.020	205.88	2383.54
	11	−1580.50^*∗*^	555.03	0.004	−2669.32	−491.67
	12	3826.02^*∗*^	555.03	0.000	2737.19	4914.85
	13	192.10	555.03	0.729	−896.72	1280.93
	14	−2743.03^*∗*^	555.03	0.000	−3831.86	−1654.20
	15	2775.18^*∗*^	555.03	0.000	1686.35	3864.01
	16	2390.32^*∗*^	555.03	0.000	1301.49	3479.15
	17	−425.99	555.03	0.443	−1514.82	662.84
	18	4870.73^*∗*^	555.03	0.000	3781.90	5959.56
	19	933.14	555.03	0.093	−155.69	2021.97
	20	−1909.87^*∗*^	555.03	0.001	−2998.69	−821.04
	21	3445.15^*∗*^	555.03	0.000	2356.32	4533.98
	22	−154.90	555.03	0.780	−1243.72	933.93
	23	−3054.05^*∗*^	555.03	0.000	−4142.87	−1965.22
	24	2405.84^*∗*^	555.03	0.000	1317.01	3494.67
	25	2014.69^*∗*^	555.03	0.000	925.86	3103.52
	26	−773.09	555.03	0.164	−1861.91	315.74
	27	4478.72^*∗*^	555.03	0.000	3389.89	5567.55

5	1	4016.24^*∗*^	555.03	0.000	2927.41	5105.07
	2	1153.62^*∗*^	555.03	0.038	64.79	2242.45
	3	6540.94^*∗*^	555.03	0.000	5452.11	7629.77
	4	2920.68^*∗*^	555.03	0.000	1831.85	4009.51
	6	5495.66^*∗*^	555.03	0.000	4406.83	6584.48
	7	5105.05^*∗*^	555.03	0.000	4016.22	6193.88
	8	2299.52^*∗*^	555.03	0.000	1210.70	3388.35
	9	7580.28^*∗*^	555.03	0.000	6491.45	8669.11
	10	4215.39^*∗*^	555.03	0.000	3126.56	5304.22
	11	1340.19^*∗*^	555.03	0.016	251.36	2429.01
	12	6746.70^*∗*^	555.03	0.000	5657.87	7835.53
	13	3112.78^*∗*^	555.03	0.000	2023.96	4201.61
	14	177.65	555.03	0.749	−911.18	1266.48
	15	5695.86^*∗*^	555.03	0.000	4607.03	6784.69
	16	5311.00^*∗*^	555.03	0.000	4222.17	6399.83
	17	2494.69^*∗*^	555.03	0.000	1405.86	3583.52
	18	7791.41^*∗*^	555.03	0.000	6702.58	8880.24
	19	3853.82^*∗*^	555.03	0.000	2764.99	4942.65
	20	1010.82	555.03	0.069	−78.01	2099.64
	21	6365.83^*∗*^	555.03	0.000	5277.00	7454.66
	22	2765.78^*∗*^	555.03	0.000	1676.96	3854.61
	23	−133.36	555.03	0.810	−1222.19	955.46
	24	5326.52^*∗*^	555.03	0.000	4237.70	6415.35
	25	4935.37^*∗*^	555.03	0.000	3846.54	6024.20
	26	2147.60^*∗*^	555.03	0.000	1058.77	3236.42
	27	7399.40^*∗*^	555.03	0.000	6310.57	8488.23

6	1	−1479.42^*∗*^	555.03	0.008	−2568.25	−390.59
	2	−4342.03^*∗*^	555.03	0.000	−5430.86	−3253.21
	3	1045.28	555.03	0.060	−43.55	2134.11
	4	−2574.97^*∗*^	555.03	0.000	−3663.80	−1486.15
	5	−5495.66^*∗*^	555.03	0.000	−6584.48	−4406.83
	7	−390.61	555.03	0.482	−1479.44	698.22
	8	−3196.13^*∗*^	555.03	0.000	−4284.96	−2107.30
	9	2084.63^*∗*^	555.03	0.000	995.80	3173.46
	10	−1280.27^*∗*^	555.03	0.021	−2369.09	−191.44
	11	−4155.47^*∗*^	555.03	0.000	−5244.30	−3066.64
	12	1251.04^*∗*^	555.03	0.024	162.21	2339.87
	13	−2382.87^*∗*^	555.03	0.000	−3471.70	−1294.04
	14	−5318.01^*∗*^	555.03	0.000	−6406.83	−4229.18
	15	200.20	555.03	0.718	−888.63	1289.03
	16	−184.66	555.03	0.739	−1273.49	904.17
	17	−3000.96^*∗*^	555.03	0.000	−4089.79	−1912.13
	18	2295.76^*∗*^	555.03	0.000	1206.93	3384.58
	19	−1641.84^*∗*^	555.03	0.003	−2730.66	−553.01
	20	−4484.84^*∗*^	555.03	0.000	−5573.67	−3396.01
	21	870.18	555.03	0.117	−218.65	1959.00
	22	−2729.87^*∗*^	555.03	0.000	−3818.70	−1641.04
	23	−5629.02^*∗*^	555.03	0.000	−6717.85	−4540.19
	24	−169.13	555.03	0.761	−1257.96	919.70
	25	−560.28	555.03	0.313	−1649.11	528.55
	26	−3348.06^*∗*^	555.03	0.000	−4436.89	−2259.23
	27	1903.75^*∗*^	555.03	0.001	814.92	2992.57

7	1	−1088.81	555.03	0.050	−2177.64	0.02
	2	−3951.42^*∗*^	555.03	0.000	−5040.25	−2862.60
	3	1435.89^*∗*^	555.03	0.010	347.06	2524.72
	4	−2184.37^*∗*^	555.03	0.000	−3273.19	−1095.54
	5	−5105.05^*∗*^	555.03	0.000	−6193.88	−4016.22
	6	390.61	555.03	0.482	−698.22	1479.44
	8	−2805.52^*∗*^	555.03	0.000	−3894.35	−1716.69
	9	2475.24^*∗*^	555.03	0.000	1386.41	3564.06
	10	−889.66	555.03	0.109	−1978.49	199.17
	11	−3764.86^*∗*^	555.03	0.000	−4853.69	−2676.03
	12	1641.65^*∗*^	555.03	0.003	552.82	2730.48
	13	−1992.26^*∗*^	555.03	0.000	−3081.09	−903.43
	14	−4927.40^*∗*^	555.03	0.000	−6016.23	−3838.57
	15	590.81	555.03	0.287	−498.02	1679.64
	16	205.95	555.03	0.711	−882.88	1294.78
	17	−2610.35^*∗*^	555.03	0.000	−3699.18	−1521.52
	18	2686.36^*∗*^	555.03	0.000	1597.54	3775.19
	19	−1251.23^*∗*^	555.03	0.024	−2340.06	−162.40
	20	−4094.23^*∗*^	555.03	0.000	−5183.06	−3005.40
	21	1260.78^*∗*^	555.03	0.023	171.96	2349.61
	22	−2339.26^*∗*^	555.03	0.000	−3428.09	−1250.43
	23	−5238.41^*∗*^	555.03	0.000	−6327.24	−4149.58
	24	221.48	555.03	0.690	−867.35	1310.31
	25	−169.67	555.03	0.760	−1258.50	919.16
	26	−2957.45^*∗*^	555.03	0.000	−4046.28	−1868.62
	27	2294.35^*∗*^	555.03	0.000	1205.53	3383.18

8	1	1716.71^*∗*^	555.03	0.002	627.89	2805.54
	2	−1145.90^*∗*^	555.03	0.039	−2234.73	−57.07
	3	4241.41^*∗*^	555.03	0.000	3152.59	5330.24
	4	621.16	555.03	0.263	−467.67	1709.98
	5	−2299.52^*∗*^	555.03	0.000	−3388.35	−1210.70
	6	3196.13^*∗*^	555.03	0.000	2107.30	4284.96
	7	2805.52^*∗*^	555.03	0.000	1716.69	3894.35
	9	5280.76^*∗*^	555.03	0.000	4191.93	6369.59
	10	1915.87^*∗*^	555.03	0.001	827.04	3004.69
	11	−959.34	555.03	0.084	−2048.17	129.49
	12	4447.17^*∗*^	555.03	0.000	3358.35	5536.00
	13	813.26	555.03	0.143	−275.57	1902.09
	14	−2121.87^*∗*^	555.03	0.000	−3210.70	−1033.05
	15	3396.33^*∗*^	555.03	0.000	2307.51	4485.16
	16	3011.47^*∗*^	555.03	0.000	1922.64	4100.30
	17	195.17	555.03	0.725	−893.66	1284.00
	18	5491.89^*∗*^	555.03	0.000	4403.06	6580.71
	19	1554.30^*∗*^	555.03	0.005	465.47	2643.12
	20	−1288.71^*∗*^	555.03	0.020	−2377.54	−199.88
	21	4066.31^*∗*^	555.03	0.000	2977.48	5155.13
	22	466.26	555.03	0.401	−622.57	1555.09
	23	−2432.89^*∗*^	555.03	0.000	−3521.72	−1344.06
	24	3027.00^*∗*^	555.03	0.000	1938.17	4115.83
	25	2635.85^*∗*^	555.03	0.000	1547.02	3724.68
	26	−151.93	555.03	0.784	−1240.76	936.90
	27	5099.88^*∗*^	555.03	0.000	4011.05	6188.71

9	1	−3564.04^*∗*^	555.03	0.000	−4652.87	−2475.22
	2	−6426.66^*∗*^	555.03	0.000	−7515.49	−5337.83
	3	−1039.34	555.03	0.061	−2128.17	49.48
	4	−4659.60^*∗*^	555.03	0.000	−5748.43	−3570.77
	5	−7580.28^*∗*^	555.03	0.000	−8669.11	−6491.45
	6	−2084.63^*∗*^	555.03	0.000	−3173.46	−995.80
	7	−2475.24^*∗*^	555.03	0.000	−3564.06	−1386.41
	8	−5280.76^*∗*^	555.03	0.000	−6369.59	−4191.93
	10	−3364.89^*∗*^	555.03	0.000	−4453.72	−2276.06
	11	−6240.10^*∗*^	555.03	0.000	−7328.93	−5151.27
	12	−833.58	555.03	0.133	−1922.41	255.25
	13	−4467.50^*∗*^	555.03	0.000	−5556.33	−3378.67
	14	−7402.63^*∗*^	555.03	0.000	−8491.46	−6313.80
	15	−1884.42^*∗*^	555.03	0.001	−2973.25	−795.59
	16	−2269.29^*∗*^	555.03	0.000	−3358.11	−1180.46
	17	−5085.59^*∗*^	555.03	0.000	−6174.42	−3996.76
	18	211.13	555.03	0.704	−877.70	1299.96
	19	−3726.46^*∗*^	555.03	0.000	−4815.29	−2637.63
	20	−6569.47^*∗*^	555.03	0.000	−7658.30	−5480.64
	21	−1214.45^*∗*^	555.03	0.029	−2303.28	−125.62
	22	−4814.50^*∗*^	555.03	0.000	−5903.33	−3725.67
	23	−7713.65^*∗*^	555.03	0.000	−8802.48	−6624.82
	24	−2253.76^*∗*^	555.03	0.000	−3342.59	−1164.93
	25	−2644.91^*∗*^	555.03	0.000	−3733.74	−1556.08
	26	−5432.69^*∗*^	555.03	0.000	−6521.52	−4343.86
	27	−180.88	555.03	0.745	−1269.71	907.95

10	1	−199.15	555.03	0.720	−1287.98	889.68
	2	−3061.77^*∗*^	555.03	0.000	−4150.60	−1972.94
	3	2325.55^*∗*^	555.03	0.000	1236.72	3414.38
	4	−1294.71^*∗*^	555.03	0.020	−2383.54	−205.88
	5	−4215.39^*∗*^	555.03	0.000	−5304.22	−3126.56
	6	1280.27^*∗*^	555.03	0.021	191.44	2369.09
	7	889.66	555.03	0.109	−199.17	1978.49
	8	−1915.87^*∗*^	555.03	0.001	−3004.69	−827.04
	9	3364.89^*∗*^	555.03	0.000	2276.06	4453.72
	11	−2875.20^*∗*^	555.03	0.000	−3964.03	−1786.38
	12	2531.31^*∗*^	555.03	0.000	1442.48	3620.14
	13	−1102.61^*∗*^	555.03	0.047	−2191.43	−13.78
	14	−4037.74^*∗*^	555.03	0.000	−5126.57	−2948.91
	15	1480.47^*∗*^	555.03	0.008	391.64	2569.30
	16	1095.61^*∗*^	555.03	0.049	6.78	2184.44
	17	−1720.70^*∗*^	555.03	0.002	−2809.53	−631.87
	18	3576.02^*∗*^	555.03	0.000	2487.19	4664.85
	19	−361.57	555.03	0.515	−1450.40	727.26
	20	−3204.57^*∗*^	555.03	0.000	−4293.40	−2115.75
	21	2150.44^*∗*^	555.03	0.000	1061.61	3239.27
	22	−1449.61^*∗*^	555.03	0.009	−2538.43	−360.78
	23	−4348.75^*∗*^	555.03	0.000	−5437.58	−3259.93
	24	1111.13^*∗*^	555.03	0.045	22.31	2199.96
	25	719.98	555.03	0.195	−368.85	1808.81
	26	−2067.79^*∗*^	555.03	0.000	−3156.62	−978.97
	27	3184.01^*∗*^	555.03	0.000	2095.18	4272.84

11	1	2676.05^*∗*^	555.03	0.000	1587.22	3764.88
	2	−186.56	555.03	0.737	−1275.39	902.26
	3	5200.75^*∗*^	555.03	0.000	4111.92	6289.58
	4	1580.50^*∗*^	555.03	0.004	491.67	2669.32
	5	−1340.19^*∗*^	555.03	0.016	−2429.01	−251.36
	6	4155.47^*∗*^	555.03	0.000	3066.64	5244.30
	7	3764.86^*∗*^	555.03	0.000	2676.03	4853.69
	8	959.34	555.03	0.084	−129.49	2048.17
	9	6240.10^*∗*^	555.03	0.000	5151.27	7328.93
	10	2875.20^*∗*^	555.03	0.000	1786.38	3964.03
	12	5406.51^*∗*^	555.03	0.000	4317.68	6495.34
	13	1772.60^*∗*^	555.03	0.001	683.77	2861.43
	14	−1162.54^*∗*^	555.03	0.036	−2251.36	−73.71
	15	4355.67^*∗*^	555.03	0.000	3266.84	5444.50
	16	3970.81^*∗*^	555.03	0.000	2881.98	5059.64
	17	1154.51^*∗*^	555.03	0.038	65.68	2243.34
	18	6451.23^*∗*^	555.03	0.000	5362.40	7540.05
	19	2513.63^*∗*^	555.03	0.000	1424.81	3602.46
	20	−329.37	555.03	0.553	−1418.20	759.46
	21	5025.65^*∗*^	555.03	0.000	3936.82	6114.47
	22	1425.60^*∗*^	555.03	0.010	336.77	2514.43
	23	−1473.55^*∗*^	555.03	0.008	−2562.38	−384.72
	24	3986.34^*∗*^	555.03	0.000	2897.51	5075.17
	25	3595.19^*∗*^	555.03	0.000	2506.36	4684.02
	26	807.41	555.03	0.146	−281.42	1896.24
	27	6059.22^*∗*^	555.03	0.000	4970.39	7148.04

12	1	−2730.46^*∗*^	555.03	0.000	−3819.29	−1641.63
	2	−5593.08^*∗*^	555.03	0.000	−6681.91	−4504.25
	3	−205.76	555.03	0.711	−1294.59	883.07
	4	−3826.02^*∗*^	555.03	0.000	−4914.85	−2737.19
	5	−6746.70^*∗*^	555.03	0.000	−7835.53	−5657.87
	6	−1251.04^*∗*^	555.03	0.024	−2339.87	−162.21
	7	−1641.65^*∗*^	555.03	0.003	−2730.48	−552.82
	8	−4447.17^*∗*^	555.03	0.000	−5536.00	−3358.35
	9	833.58	555.03	0.133	−255.25	1922.41
	10	−2531.31^*∗*^	555.03	0.000	−3620.14	−1442.48
	11	−5406.51^*∗*^	555.03	0.000	−6495.34	−4317.68
	13	−3633.91^*∗*^	555.03	0.000	−4722.74	−2545.09
	14	−6569.05^*∗*^	555.03	0.000	−7657.88	−5480.22
	15	−1050.84	555.03	0.059	−2139.67	37.99
	16	−1435.70^*∗*^	555.03	0.010	−2524.53	−346.87
	17	−4252.01^*∗*^	555.03	0.000	−5340.83	−3163.18
	18	1044.71	555.03	0.060	−44.12	2133.54
	19	−2892.88^*∗*^	555.03	0.000	−3981.71	−1804.05
	20	−5735.88^*∗*^	555.03	0.000	−6824.71	−4647.06
	21	−380.87	555.03	0.493	−1469.70	707.96
	22	−3980.91^*∗*^	555.03	0.000	−5069.74	−2892.09
	23	−6880.06^*∗*^	555.03	0.000	−7968.89	−5791.24
	24	−1420.18^*∗*^	555.03	0.011	−2509.00	−331.35
	25	−1811.33^*∗*^	555.03	0.001	−2900.15	−722.50
	26	−4599.10^*∗*^	555.03	0.000	−5687.93	−3510.28
	27	652.70	555.03	0.240	−436.13	1741.53

13	1	903.45	555.03	0.104	−185.37	1992.28
	2	−1959.16^*∗*^	555.03	0.000	−3047.99	−870.33
	3	3428.15^*∗*^	555.03	0.000	2339.33	4516.98
	4	−192.10	555.03	0.729	−1280.93	896.72
	5	−3112.78^*∗*^	555.03	0.000	−4201.61	−2023.96
	6	2382.87^*∗*^	555.03	0.000	1294.04	3471.70
	7	1992.26^*∗*^	555.03	0.000	903.43	3081.09
	8	−813.26	555.03	0.143	−1902.09	275.57
	9	4467.50^*∗*^	555.03	0.000	3378.67	5556.33
	10	1102.61^*∗*^	555.03	0.047	13.78	2191.43
	11	−1772.60^*∗*^	555.03	0.001	−2861.43	−683.77
	12	3633.91^*∗*^	555.03	0.000	2545.09	4722.74
	14	−2935.13^*∗*^	555.03	0.000	−4023.96	−1846.31
	15	2583.07^*∗*^	555.03	0.000	1494.25	3671.90
	16	2198.21^*∗*^	555.03	0.000	1109.38	3287.04
	17	−618.09	555.03	0.266	−1706.92	470.74
	18	4678.63^*∗*^	555.03	0.000	3589.80	5767.45
	19	741.04	555.03	0.182	−347.79	1829.86
	20	−2101.97^*∗*^	555.03	0.000	−3190.80	−1013.14
	21	3253.05^*∗*^	555.03	0.000	2164.22	4341.87
	22	−347.00	555.03	0.532	−1435.83	741.83
	23	−3246.15^*∗*^	555.03	0.000	−4334.98	−2157.32
	24	2213.74^*∗*^	555.03	0.000	1124.91	3302.57
	25	1822.59^*∗*^	555.03	0.001	733.76	2911.42
	26	−965.19	555.03	0.082	−2054.02	123.64
	27	4286.62^*∗*^	555.03	0.000	3197.79	5375.45

14	1	3838.59^*∗*^	555.03	0.000	2749.76	4927.42
	2	975.97	555.03	0.079	−112.86	2064.80
	3	6363.29^*∗*^	555.03	0.000	5274.46	7452.12
	4	2743.03^*∗*^	555.03	0.000	1654.20	3831.86
	5	−177.65	555.03	0.749	−1266.48	911.18
	6	5318.01^*∗*^	555.03	0.000	4229.18	6406.83
	7	4927.40^*∗*^	555.03	0.000	3838.57	6016.23
	8	2121.87^*∗*^	555.03	0.000	1033.05	3210.70
	9	7402.63^*∗*^	555.03	0.000	6313.80	8491.46
	10	4037.74^*∗*^	555.03	0.000	2948.91	5126.57
	11	1162.54^*∗*^	555.03	0.036	73.71	2251.36
	12	6569.05^*∗*^	555.03	0.000	5480.22	7657.88
	13	2935.13^*∗*^	555.03	0.000	1846.31	4023.96
	15	5518.21^*∗*^	555.03	0.000	4429.38	6607.04
	16	5133.35^*∗*^	555.03	0.000	4044.52	6222.18
	17	2317.04^*∗*^	555.03	0.000	1228.21	3405.87
	18	7613.76^*∗*^	555.03	0.000	6524.93	8702.59
	19	3676.17^*∗*^	555.03	0.000	2587.34	4765.00
	20	833.17	555.03	0.134	−255.66	1921.99
	21	6188.18^*∗*^	555.03	0.000	5099.35	7277.01
	22	2588.13^*∗*^	555.03	0.000	1499.31	3676.96
	23	−311.01	555.03	0.575	−1399.84	777.81
	24	5148.87^*∗*^	555.03	0.000	4060.05	6237.70
	25	4757.72^*∗*^	555.03	0.000	3668.89	5846.55
	26	1969.95^*∗*^	555.03	0.000	881.12	3058.77
	27	7221.75^*∗*^	555.03	0.000	6132.92	8310.58

15	1	−1679.62^*∗*^	555.03	0.003	−2768.45	−590.79
	2	−4542.24^*∗*^	555.03	0.000	−5631.07	−3453.41
	3	845.08	555.03	0.128	−243.75	1933.91
	4	−2775.18^*∗*^	555.03	0.000	−3864.01	−1686.35
	5	−5695.86^*∗*^	555.03	0.000	−6784.69	−4607.03
	6	−200.20	555.03	0.718	−1289.03	888.63
	7	−590.81	555.03	0.287	−1679.64	498.02
	8	−3396.33^*∗*^	555.03	0.000	−4485.16	−2307.51
	9	1884.42^*∗*^	555.03	0.001	795.59	2973.25
	10	−1480.47^*∗*^	555.03	0.008	−2569.30	−391.64
	11	−4355.67^*∗*^	555.03	0.000	−5444.50	−3266.84
	12	1050.84	555.03	0.059	−37.99	2139.67
	13	−2583.07^*∗*^	555.03	0.000	−3671.90	−1494.25
	14	−5518.21^*∗*^	555.03	0.000	−6607.04	−4429.38
	16	−384.86	555.03	0.488	−1473.69	703.97
	17	−3201.17^*∗*^	555.03	0.000	−4289.99	−2112.34
	18	2095.55^*∗*^	555.03	0.000	1006.72	3184.38
	19	−1842.04^*∗*^	555.03	0.001	−2930.87	−753.21
	20	−4685.04^*∗*^	555.03	0.000	−5773.87	−3596.22
	21	669.97	555.03	0.228	−418.86	1758.80
	22	−2930.07^*∗*^	555.03	0.000	−4018.90	−1841.25
	23	−5829.22^*∗*^	555.03	0.000	−6918.05	−4740.40
	24	−369.34	555.03	0.506	−1458.16	719.49
	25	−760.49	555.03	0.171	−1849.31	328.34
	26	−3548.26^*∗*^	555.03	0.000	−4637.09	−2459.44
	27	1703.54^*∗*^	555.03	0.002	614.71	2792.37

16	1	−1294.76^*∗*^	555.03	0.020	−2383.59	−205.93
	2	−4157.38^*∗*^	555.03	0.000	−5246.20	−3068.55
	3	1229.94^*∗*^	555.03	0.027	141.11	2318.77
	4	−2390.32^*∗*^	555.03	0.000	−3479.15	−1301.49
	5	−5311.00^*∗*^	555.03	0.000	−6399.83	−4222.17
	6	184.66	555.03	0.739	−904.17	1273.49
	7	−205.95	555.03	0.711	−1294.78	882.88
	8	−3011.47^*∗*^	555.03	0.000	−4100.30	−1922.64
	9	2269.29^*∗*^	555.03	0.000	1180.46	3358.11
	10	−1095.61^*∗*^	555.03	0.049	−2184.44	−6.78
	11	−3970.81^*∗*^	555.03	0.000	−5059.64	−2881.98
	12	1435.70^*∗*^	555.03	0.010	346.87	2524.53
	13	−2198.21^*∗*^	555.03	0.000	−3287.04	−1109.38
	14	−5133.35^*∗*^	555.03	0.000	−6222.18	−4044.52
	15	384.86	555.03	0.488	−703.97	1473.69
	17	−2816.30^*∗*^	555.03	0.000	−3905.13	−1727.48
	18	2480.41^*∗*^	555.03	0.000	1391.58	3569.24
	19	−1457.18^*∗*^	555.03	0.009	−2546.01	−368.35
	20	−4300.18^*∗*^	555.03	0.000	−5389.01	−3211.35
	21	1054.83	555.03	0.058	−34.00	2143.66
	22	−2545.21^*∗*^	555.03	0.000	−3634.04	−1456.38
	23	−5444.36^*∗*^	555.03	0.000	−6533.19	−4355.53
	24	15.53	555.03	0.978	−1073.30	1104.36
	25	−375.62	555.03	0.499	−1464.45	713.20
	26	−3163.40^*∗*^	555.03	0.000	−4252.23	−2074.57
	27	2088.40^*∗*^	555.03	0.000	999.58	3177.23

17	1	1521.55^*∗*^	555.03	0.006	432.72	2610.37
	2	−1341.07^*∗*^	555.03	0.016	−2429.90	−252.24
	3	4046.25^*∗*^	555.03	0.000	2957.42	5135.07
	4	425.99	555.03	0.443	−662.84	1514.82
	5	−2494.69^*∗*^	555.03	0.000	−3583.52	−1405.86
	6	3000.96^*∗*^	555.03	0.000	1912.13	4089.79
	7	2610.35^*∗*^	555.03	0.000	1521.52	3699.18
	8	−195.17	555.03	0.725	−1284.00	893.66
	9	5085.59^*∗*^	555.03	0.000	3996.76	6174.42
	10	1720.70^*∗*^	555.03	0.002	631.87	2809.53
	11	−1154.51^*∗*^	555.03	0.038	−2243.34	−65.68
	12	4252.01^*∗*^	555.03	0.000	3163.18	5340.83
	13	618.09	555.03	0.266	−470.74	1706.92
	14	−2317.04^*∗*^	555.03	0.000	−3405.87	−1228.21
	15	3201.17^*∗*^	555.03	0.000	2112.34	4289.99
	16	2816.30^*∗*^	555.03	0.000	1727.48	3905.13
	18	5296.72^*∗*^	555.03	0.000	4207.89	6385.55
	19	1359.13^*∗*^	555.03	0.014	270.30	2447.96
	20	−1483.88^*∗*^	555.03	0.008	−2572.71	−395.05
	21	3871.14^*∗*^	555.03	0.000	2782.31	4959.97
	22	271.09	555.03	0.625	−817.74	1359.92
	23	−2628.06^*∗*^	555.03	0.000	−3716.89	−1539.23
	24	2831.83^*∗*^	555.03	0.000	1743.00	3920.66
	25	2440.68^*∗*^	555.03	0.000	1351.85	3529.51
	26	−347.10	555.03	0.532	−1435.93	741.73
	27	4904.71^*∗*^	555.03	0.000	3815.88	5993.54

18	1	−3775.17^*∗*^	555.03	0.000	−4864.00	−2686.34
	2	−6637.79^*∗*^	555.03	0.000	−7726.62	−5548.96
	3	−1250.47^*∗*^	555.03	0.024	−2339.30	−161.64
	4	−4870.73^*∗*^	555.03	0.000	−5959.56	−3781.90
	5	−7791.41^*∗*^	555.03	0.000	−8880.24	−6702.58
	6	−2295.76^*∗*^	555.03	0.000	−3384.58	−1206.93
	7	−2686.36^*∗*^	555.03	0.000	−3775.19	−1597.54
	8	−5491.89^*∗*^	555.03	0.000	−6580.71	−4403.06
	9	−211.13	555.03	0.704	−1299.96	877.70
	10	−3576.02^*∗*^	555.03	0.000	−4664.85	−2487.19
	11	−6451.23^*∗*^	555.03	0.000	−7540.05	−5362.40
	12	−1044.71	555.03	0.060	−2133.54	44.12
	13	−4678.63^*∗*^	555.03	0.000	−5767.45	−3589.80
	14	−7613.76^*∗*^	555.03	0.000	−8702.59	−6524.93
	15	−2095.55^*∗*^	555.03	0.000	−3184.38	−1006.72
	16	−2480.41^*∗*^	555.03	0.000	−3569.24	−1391.58
	17	−5296.72^*∗*^	555.03	0.000	−6385.55	−4207.89
	19	−3937.59^*∗*^	555.03	0.000	−5026.42	−2848.76
	20	−6780.60^*∗*^	555.03	0.000	−7869.42	−5691.77
	21	−1425.58^*∗*^	555.03	0.010	−2514.41	−336.75
	22	−5025.63^*∗*^	555.03	0.000	−6114.45	−3936.80
	23	−7924.78^*∗*^	555.03	0.000	−9013.60	−6835.95
	24	−2464.89^*∗*^	555.03	0.000	−3553.72	−1376.06
	25	−2856.04^*∗*^	555.03	0.000	−3944.87	−1767.21
	26	−5643.82^*∗*^	555.03	0.000	−6732.64	−4554.99
	27	−392.01	555.03	0.480	−1480.84	696.82

19	1	162.42	555.03	0.770	−926.41	1251.25
	2	−2700.20^*∗*^	555.03	0.000	−3789.03	−1611.37
	3	2687.12^*∗*^	555.03	0.000	1598.29	3775.95
	4	−933.14	555.03	0.093	−2021.97	155.69
	5	−3853.82^*∗*^	555.03	0.000	−4942.65	−2764.99
	6	1641.84^*∗*^	555.03	0.003	553.01	2730.66
	7	1251.23^*∗*^	555.03	0.024	162.40	2340.06
	8	−1554.30^*∗*^	555.03	0.005	−2643.12	−465.47
	9	3726.46^*∗*^	555.03	0.000	2637.63	4815.29
	10	361.57	555.03	0.515	−727.26	1450.40
	11	−2513.63^*∗*^	555.03	0.000	−3602.46	−1424.81
	12	2892.88^*∗*^	555.03	0.000	1804.05	3981.71
	13	−741.04	555.03	0.182	−1829.86	347.79
	14	−3676.17^*∗*^	555.03	0.000	−4765.00	−2587.34
	15	1842.04^*∗*^	555.03	0.001	753.21	2930.87
	16	1457.18^*∗*^	555.03	0.009	368.35	2546.01
	17	−1359.13^*∗*^	555.03	0.014	−2447.96	−270.30
	18	3937.59^*∗*^	555.03	0.000	2848.76	5026.42
	20	−2843.00^*∗*^	555.03	0.000	−3931.83	−1754.18
	21	2512.01^*∗*^	555.03	0.000	1423.18	3600.84
	22	−1088.04	555.03	0.050	−2176.86	.79
	23	−3987.18^*∗*^	555.03	0.000	−5076.01	−2898.36
	24	1472.70^*∗*^	555.03	0.008	383.88	2561.53
	25	1081.55	555.03	0.052	−7.28	2170.38
	26	−1706.22^*∗*^	555.03	0.002	−2795.05	−617.40
	27	3545.58^*∗*^	555.03	0.000	2456.75	4634.41

20	1	3005.42^*∗*^	555.03	0.000	1916.59	4094.25
	2	142.81	555.03	0.797	−946.02	1231.63
	3	5530.12^*∗*^	555.03	0.000	4441.29	6618.95
	4	1909.87^*∗*^	555.03	0.001	821.04	2998.69
	5	−1010.82	555.03	0.069	−2099.64	78.01
	6	4484.84^*∗*^	555.03	0.000	3396.01	5573.67
	7	4094.23^*∗*^	555.03	0.000	3005.40	5183.06
	8	1288.71^*∗*^	555.03	0.020	199.88	2377.54
	9	6569.47^*∗*^	555.03	0.000	5480.64	7658.30
	10	3204.57^*∗*^	555.03	0.000	2115.75	4293.40
	11	329.37	555.03	0.553	−759.46	1418.20
	12	5735.88^*∗*^	555.03	0.000	4647.06	6824.71
	13	2101.97^*∗*^	555.03	0.000	1013.14	3190.80
	14	−833.17	555.03	0.134	−1921.99	255.66
	15	4685.04^*∗*^	555.03	0.000	3596.22	5773.87
	16	4300.18^*∗*^	555.03	0.000	3211.35	5389.01
	17	1483.88^*∗*^	555.03	0.008	395.05	2572.71
	18	6780.60^*∗*^	555.03	0.000	5691.77	7869.42
	19	2843.00^*∗*^	555.03	0.000	1754.18	3931.83
	21	5355.02^*∗*^	555.03	0.000	4266.19	6443.84
	22	1754.97^*∗*^	555.03	0.002	666.14	2843.80
	23	−1144.18^*∗*^	555.03	0.039	−2233.01	−55.35
	24	4315.71^*∗*^	555.03	0.000	3226.88	5404.54
	25	3924.56^*∗*^	555.03	0.000	2835.73	5013.39
	26	1136.78^*∗*^	555.03	0.041	47.95	2225.61
	27	6388.59^*∗*^	555.03	0.000	5299.76	7477.41

21	1	−2349.59^*∗*^	555.03	0.000	−3438.42	−1260.76
	2	−5212.21^*∗*^	555.03	0.000	−6301.04	−4123.38
	3	175.11	555.03	0.752	−913.72	1263.94
	4	−3445.15^*∗*^	555.03	0.000	−4533.98	−2356.32
	5	−6365.83^*∗*^	555.03	0.000	−7454.66	−5277.00
	6	−870.18	555.03	0.117	−1959.00	218.65
	7	−1260.78^*∗*^	555.03	0.023	−2349.61	−171.96
	8	−4066.31^*∗*^	555.03	0.000	−5155.13	−2977.48
	9	1214.45^*∗*^	555.03	0.029	125.62	2303.28
	10	−2150.44^*∗*^	555.03	0.000	−3239.27	−1061.61
	11	−5025.65^*∗*^	555.03	0.000	−6114.47	−3936.82
	12	380.87	555.03	0.493	−707.96	1469.70
	13	−3253.05^*∗*^	555.03	0.000	−4341.87	−2164.22
	14	−6188.18^*∗*^	555.03	0.000	−7277.01	−5099.35
	15	−669.97	555.03	0.228	−1758.80	418.86
	16	−1054.83	555.03	0.058	−2143.66	34.00
	17	−3871.14^*∗*^	555.03	0.000	−4959.97	−2782.31
	18	1425.58^*∗*^	555.03	0.010	336.75	2514.41
	19	−2512.01^*∗*^	555.03	0.000	−3600.84	−1423.18
	20	−5355.02^*∗*^	555.03	0.000	−6443.84	−4266.19
	22	−3600.05^*∗*^	555.03	0.000	−4688.87	−2511.22
	23	−6499.20^*∗*^	555.03	0.000	−7588.02	−5410.37
	24	−1039.31	555.03	0.061	−2128.14	49.52
	25	−1430.46^*∗*^	555.03	0.010	−2519.29	−341.63
	26	−4218.24^*∗*^	555.03	0.000	−5307.06	−3129.41
	27	1033.57	555.03	0.063	−55.26	2122.40

22	1	1250.45^*∗*^	555.03	0.024	161.63	2339.28
	2	−1612.16^*∗*^	555.03	0.004	−2700.99	−523.33
	3	3775.15^*∗*^	555.03	0.000	2686.33	4863.98
	4	154.90	555.03	0.780	−933.93	1243.72
	5	−2765.78^*∗*^	555.03	0.000	−3854.61	−1676.96
	6	2729.87^*∗*^	555.03	0.000	1641.04	3818.70
	7	2339.26^*∗*^	555.03	0.000	1250.43	3428.09
	8	−466.26	555.03	0.401	−1555.09	622.57
	9	4814.50^*∗*^	555.03	0.000	3725.67	5903.33
	10	1449.61^*∗*^	555.03	0.009	360.78	2538.43
	11	−1425.60^*∗*^	555.03	0.010	−2514.43	−336.77
	12	3980.91^*∗*^	555.03	0.000	2892.09	5069.74
	13	347.00	555.03	0.532	−741.83	1435.83
	14	−2588.13^*∗*^	555.03	0.000	−3676.96	−1499.31
	15	2930.07^*∗*^	555.03	0.000	1841.25	4018.90
	16	2545.21^*∗*^	555.03	0.000	1456.38	3634.04
	17	−271.09	555.03	0.625	−1359.92	817.74
	18	5025.63^*∗*^	555.03	0.000	3936.80	6114.45
	19	1088.04	555.03	0.050	−0.79	2176.86
	20	−1754.97^*∗*^	555.03	0.002	−2843.80	−666.14
	21	3600.05^*∗*^	555.03	0.000	2511.22	4688.87
	23	−2899.15^*∗*^	555.03	0.000	−3987.98	−1810.32
	24	2560.74^*∗*^	555.03	0.000	1471.91	3649.57
	25	2169.59^*∗*^	555.03	0.000	1080.76	3258.42
	26	−618.19	555.03	0.266	−1707.02	470.64
	27	4633.62^*∗*^	555.03	0.000	3544.79	5722.45

23	1	4149.60^*∗*^	555.03	0.000	3060.77	5238.43
	2	1286.99^*∗*^	555.03	0.021	198.16	2375.81
	3	6674.30^*∗*^	555.03	0.000	5585.47	7763.13
	4	3054.05^*∗*^	555.03	0.000	1965.22	4142.87
	5	133.36	555.03	0.810	−955.46	1222.19
	6	5629.02^*∗*^	555.03	0.000	4540.19	6717.85
	7	5238.41^*∗*^	555.03	0.000	4149.58	6327.24
	8	2432.89^*∗*^	555.03	0.000	1344.06	3521.72
	9	7713.65^*∗*^	555.03	0.000	6624.82	8802.48
	10	4348.75^*∗*^	555.03	0.000	3259.93	5437.58
	11	1473.55^*∗*^	555.03	0.008	384.72	2562.38
	12	6880.06^*∗*^	555.03	0.000	5791.24	7968.89
	13	3246.15^*∗*^	555.03	0.000	2157.32	4334.98
	14	311.01	555.03	0.575	−777.81	1399.84
	15	5829.22^*∗*^	555.03	0.000	4740.40	6918.05
	16	5444.36^*∗*^	555.03	0.000	4355.53	6533.19
	17	2628.06^*∗*^	555.03	0.000	1539.23	3716.89
	18	7924.78^*∗*^	555.03	0.000	6835.95	9013.60
	19	3987.18^*∗*^	555.03	0.000	2898.36	5076.01
	20	1144.18^*∗*^	555.03	0.039	55.35	2233.01
	21	6499.20^*∗*^	555.03	0.000	5410.37	7588.02
	22	2899.15^*∗*^	555.03	0.000	1810.32	3987.98
	24	5459.89^*∗*^	555.03	0.000	4371.06	6548.72
	25	5068.74^*∗*^	555.03	0.000	3979.91	6157.57
	26	2280.96^*∗*^	555.03	0.000	1192.13	3369.79
	27	7532.77^*∗*^	555.03	0.000	6443.94	8621.59

24	1	−1310.29^*∗*^	555.03	0.018	−2399.11	−221.46
	2	−4172.90^*∗*^	555.03	0.000	−5261.73	−3084.07
	3	1214.41^*∗*^	555.03	0.029	125.59	2303.24
	4	−2405.84^*∗*^	555.03	0.000	−3494.67	−1317.01
	5	−5326.52^*∗*^	555.03	0.000	−6415.35	−4237.70
	6	169.13	555.03	0.761	−919.70	1257.96
	7	−221.48	555.03	0.690	−1310.31	867.35
	8	−3027.00^*∗*^	555.03	0.000	−4115.83	−1938.17
	9	2253.76^*∗*^	555.03	0.000	1164.93	3342.59
	10	−1111.13^*∗*^	555.03	0.045	−2199.96	−22.31
	11	−3986.34^*∗*^	555.03	0.000	−5075.17	−2897.51
	12	1420.18^*∗*^	555.03	0.011	331.35	2509.00
	13	−2213.74^*∗*^	555.03	0.000	−3302.57	−1124.91
	14	−5148.87^*∗*^	555.03	0.000	−6237.70	−4060.05
	15	369.34	555.03	0.506	−719.49	1458.16
	16	−15.53	555.03	0.978	−1104.36	1073.30
	17	−2831.83^*∗*^	555.03	0.000	−3920.66	−1743.00
	18	2464.89^*∗*^	555.03	0.000	1376.06	3553.72
	19	−1472.70^*∗*^	555.03	0.008	−2561.53	−383.88
	20	−4315.71^*∗*^	555.03	0.000	−5404.54	−3226.88
	21	1039.31	555.03	0.061	−49.52	2128.14
	22	−2560.74^*∗*^	555.03	0.000	−3649.57	−1471.91
	23	−5459.89^*∗*^	555.03	0.000	−6548.72	−4371.06
	25	−391.15	555.03	0.481	−1479.98	697.68
	26	−3178.93^*∗*^	555.03	0.000	−4267.76	−2090.10
	27	2072.88^*∗*^	555.03	0.000	984.05	3161.71

25	1	−919.13	555.03	0.098	−2007.96	169.69
	2	−3781.75^*∗*^	555.03	0.000	−4870.58	−2692.92
	3	1605.57^*∗*^	555.03	0.004	516.74	2694.39
	4	−2014.69^*∗*^	555.03	0.000	−3103.52	−925.86
	5	−4935.37^*∗*^	555.03	0.000	−6024.20	−3846.54
	6	560.28	555.03	0.313	−528.55	1649.11
	7	169.67	555.03	0.760	−919.16	1258.50
	8	−2635.85^*∗*^	555.03	0.000	−3724.68	−1547.02
	9	2644.91^*∗*^	555.03	0.000	1556.08	3733.74
	10	−719.98	555.03	0.195	−1808.81	368.85
	11	−3595.19^*∗*^	555.03	0.000	−4684.02	−2506.36
	12	1811.33^*∗*^	555.03	0.001	722.50	2900.15
	13	−1822.59^*∗*^	555.03	0.001	−2911.42	−733.76
	14	−4757.72^*∗*^	555.03	0.000	−5846.55	−3668.89
	15	760.49	555.03	0.171	−328.34	1849.31
	16	375.62	555.03	0.499	−713.20	1464.45
	17	−2440.68^*∗*^	555.03	0.000	−3529.51	−1351.85
	18	2856.04^*∗*^	555.03	0.000	1767.21	3944.87
	19	−1081.55	555.03	0.052	−2170.38	7.28
	20	−3924.56^*∗*^	555.03	0.000	−5013.39	−2835.73
	21	1430.46^*∗*^	555.03	0.010	341.63	2519.29
	22	−2169.59^*∗*^	555.03	0.000	−3258.42	−1080.76
	23	−5068.74^*∗*^	555.03	0.000	−6157.57	−3979.91
	24	391.15	555.03	0.481	−697.68	1479.98
	26	−2787.78^*∗*^	555.03	0.000	−3876.61	−1698.95
	27	2464.03^*∗*^	555.03	0.000	1375.20	3552.86

26	1	1868.64^*∗*^	555.03	0.001	779.81	2957.47
	2	−993.97	555.03	0.074	−2082.80	94.86
	3	4393.34^*∗*^	555.03	0.000	3304.51	5482.17
	4	773.09	555.03	0.164	−315.74	1861.91
	5	−2147.60^*∗*^	555.03	0.000	−3236.42	−1058.77
	6	3348.06^*∗*^	555.03	0.000	2259.23	4436.89
	7	2957.45^*∗*^	555.03	0.000	1868.62	4046.28
	8	151.93	555.03	0.784	−936.90	1240.76
	9	5432.69^*∗*^	555.03	0.000	4343.86	6521.52
	10	2067.79^*∗*^	555.03	0.000	978.97	3156.62
	11	−807.41	555.03	0.146	−1896.24	281.42
	12	4599.10^*∗*^	555.03	0.000	3510.28	5687.93
	13	965.19	555.03	0.082	−123.64	2054.02
	14	−1969.95^*∗*^	555.03	0.000	−3058.77	−881.12
	15	3548.26^*∗*^	555.03	0.000	2459.44	4637.09
	16	3163.40^*∗*^	555.03	0.000	2074.57	4252.23
	17	347.10	555.03	0.532	−741.73	1435.93
	18	5643.82^*∗*^	555.03	0.000	4554.99	6732.64
	19	1706.22^*∗*^	555.03	0.002	617.40	2795.05
	20	−1136.78^*∗*^	555.03	0.041	−2225.61	−47.95
	21	4218.24^*∗*^	555.03	0.000	3129.41	5307.06
	22	618.19	555.03	0.266	−470.64	1707.02
	23	−2280.96^*∗*^	555.03	0.000	−3369.79	−1192.13
	24	3178.93^*∗*^	555.03	0.000	2090.10	4267.76
	25	2787.78^*∗*^	555.03	0.000	1698.95	3876.61
	27	5251.81^*∗*^	555.03	0.000	4162.98	6340.63

27	1	−3383.16^*∗*^	555.03	0.000	−4471.99	−2294.33
	2	−6245.78^*∗*^	555.03	0.000	−7334.61	−5156.95
	3	−858.46	555.03	0.122	−1947.29	230.37
	4	−4478.72^*∗*^	555.03	0.000	−5567.55	−3389.89
	5	−7399.40^*∗*^	555.03	0.000	−8488.23	−6310.57
	6	−1903.75^*∗*^	555.03	0.001	−2992.57	−814.92
	7	−2294.35^*∗*^	555.03	0.000	−3383.18	−1205.53
	8	−5099.88^*∗*^	555.03	0.000	−6188.71	−4011.05
	9	180.88	555.03	0.745	−907.95	1269.71
	10	−3184.01^*∗*^	555.03	0.000	−4272.84	−2095.18
	11	−6059.22^*∗*^	555.03	0.000	−7148.04	−4970.39
	12	−652.70	555.03	0.240	−1741.53	436.13
	13	−4286.62^*∗*^	555.03	0.000	−5375.45	−3197.79
	14	−7221.75^*∗*^	555.03	0.000	−8310.58	−6132.92
	15	−1703.54^*∗*^	555.03	0.002	−2792.37	−614.71
	16	−2088.40^*∗*^	555.03	0.000	−3177.23	−999.58
	17	−4904.71^*∗*^	555.03	0.000	−5993.54	−3815.88
	18	392.01	555.03	0.480	−696.82	1480.84
	19	−3545.58^*∗*^	555.03	0.000	−4634.41	−2456.75
	20	−6388.59^*∗*^	555.03	0.000	−7477.41	−5299.76
	21	−1033.57	555.03	0.063	−2122.40	55.26
	22	−4633.62^*∗*^	555.03	0.000	−5722.45	−3544.79
	23	−7532.77^*∗*^	555.03	0.000	−8621.59	−6443.94
	24	−2072.88^*∗*^	555.03	0.000	−3161.71	−984.05
	25	−2464.03^*∗*^	555.03	0.000	−3552.86	−1375.20
	26	−5251.81^*∗*^	555.03	0.000	−6340.63	−4162.98

^*∗*^The mean difference is significant at the 0.05 level.

**Table 7:** Welch's ANOVA.

Annual_Patent_Application_Acceptance				
	Statistic^a^	df1	df2	Sig.
Welch	141.386	26	474.820	0.000

^a^Asymptotically *F* distributed.

**Table 8:** Multiple comparisons on dependent variable Annual_Patent_Application_Acceptance.

(*I*) experiment	(*J*) experiment	Mean difference (*I* − *J*)	Std. error	Sig.	95% confidence interval	
					Lower bound	Upper bound
1	2	−1334561.15^*∗*^	130309.35	0.000	−1831489.21	−837633.09
	3	1035494.80^*∗*^	102289.44	0.000	645497.88	1425491.72
	4	−279970.62	115257.21	0.773	−718756.81	158815.57
	5	−1677613.28^*∗*^	133154.93	0.000	−2185637.58	−1169588.98
	6	803578.50^*∗*^	103540.25	0.000	408957.30	1198199.71
	7	272330.42	111560.80	0.766	−152383.64	697044.47
	8	−1001451.13^*∗*^	127580.66	0.000	−1487763.65	−515138.61
	9	1261452.03^*∗*^	101091.25	0.000	875863.92	1647040.14
	10	−5183.39	114421.05	1.000	−440777.19	430410.41
	11	−1367536.94^*∗*^	132259.72	0.000	−1872067.76	−863006.12
	12	1046576.35^*∗*^	102840.85	0.000	654543.52	1438609.19
	13	−292030.25	116426.81	0.720	−735290.17	151229.67
	14	−1720718.86^*∗*^	135305.26	0.000	−2237143.77	−1204293.95
	15	809955.73^*∗*^	104160.56	0.000	413033.36	1206878.10
	16	273579.28	112501.66	0.772	−154706.32	701864.87
	17	−1024941.70^*∗*^	129343.67	0.000	−1518109.97	−531773.43
	18	1276929.24^*∗*^	101578.27	0.000	889551.66	1664306.82
	19	19204.88	112248.20	1.000	−408117.84	446527.60
	20	−1283466.38^*∗*^	128269.37	0.000	−1772455.73	−794477.03
	21	1035215.93^*∗*^	101686.92	0.000	647438.68	1422993.18
	22	−253584.42	114019.61	0.885	−687647.34	180478.50
	23	−1616054.61^*∗*^	130920.72	0.000	−2115364.60	−1116744.62
	24	808265.88^*∗*^	102869.27	0.000	416127.98	1200403.79
	25	284792.10	110548.05	0.672	−136086.08	705670.28
	26	−960192.23^*∗*^	125723.50	0.000	−1439295.57	−481088.89
	27	1256511.66^*∗*^	100552.72	0.000	872898.01	1640125.31

2	1	1334561.15^*∗*^	130309.35	0.000	837633.09	1831489.21
	3	2370055.95^*∗*^	120793.10	0.000	1907536.83	2832575.07
	4	1054590.53^*∗*^	131954.79	0.000	551592.83	1557588.23
	5	−343052.13	147844.77	0.838	−905901.73	219797.47
	6	2138139.66^*∗*^	121854.12	0.000	1671852.25	2604427.06
	7	1606891.57^*∗*^	128738.71	0.000	1115728.06	2098055.07
	8	333110.02	142844.91	0.832	−210705.14	876925.18
	9	2596013.18^*∗*^	119780.14	0.000	2137072.98	3054953.38
	10	1329377.76^*∗*^	131225.07	0.000	829075.53	1829679.99
	11	−32975.79	147039.03	1.000	−592747.86	526796.28
	12	2381137.51^*∗*^	121260.39	0.000	1916961.17	2845313.84
	13	1042530.90^*∗*^	132977.61	0.000	535745.48	1549316.32
	14	−386157.71	149784.37	0.670	−956429.94	184114.52
	15	2144516.88^*∗*^	122381.64	0.000	1676348.80	2612684.96
	16	1608140.43^*∗*^	129554.87	0.000	1113985.11	2102295.74
	17	309619.45	144421.70	0.918	−240181.90	859420.80
	18	2611490.39^*∗*^	120191.47	0.000	2151099.19	3071881.59
	19	1353766.03^*∗*^	129334.84	0.000	860418.09	1847113.97
	20	51094.77	143460.36	1.000	−495054.97	597244.51
	21	2369777.08^*∗*^	120283.30	0.000	1909061.50	2830492.66
	22	1080976.73^*∗*^	130875.18	0.000	581964.86	1579988.60
	23	−281493.46	145835.79	0.972	−836676.72	273689.80
	24	2142827.04^*∗*^	121284.50	0.000	1678565.09	2607088.98
	25	1619353.25^*∗*^	127862.10	0.000	1131393.93	2107312.57
	26	374368.92	141188.68	0.616	−163176.58	911914.42
	27	2591072.81^*∗*^	119325.99	0.000	2133731.03	3048414.59

3	1	−1035494.80^*∗*^	102289.44	0.000	−1425491.72	−645497.88
	2	−2370055.95^*∗*^	120793.10	0.000	−2832575.07	−1907536.83
	4	−1315465.42^*∗*^	104377.54	0.000	−1713595.91	−917334.93
	5	−2713108.08^*∗*^	123857.50	0.000	−3187702.83	−2238513.33
	6	−231916.29	91274.47	0.697	−579398.77	115566.18
	7	−763164.38^*∗*^	100280.89	0.000	−1145355.72	−380973.05
	8	−2036945.93^*∗*^	117844.27	0.000	−2487856.77	−1586035.09
	9	225957.23	88486.65	0.688	−110912.12	562826.58
	10	−1040678.19^*∗*^	103453.49	0.000	−1435207.09	−646149.29
	11	−2403031.74^*∗*^	122894.59	0.000	−2873830.79	−1932232.69
	12	11081.55	90480.31	1.000	−333369.89	355533.00
	13	−1327525.05^*∗*^	105667.63	0.000	−1730689.26	−924360.84
	14	−2756213.66^*∗*^	126166.40	0.000	−3239913.82	−2272513.50
	15	−225539.07	91977.54	0.758	−575709.66	124631.52
	16	−761915.52^*∗*^	101326.55	0.000	−1148168.10	−375662.95
	17	−2060436.50^*∗*^	119750.71	0.000	−2518850.71	−1602022.29
	18	241434.44	89042.64	0.570	−97545.33	580414.21
	19	−1016289.92^*∗*^	101045.06	0.000	−1401448.69	−631131.15
	20	−2318961.18^*∗*^	118589.54	0.000	−2772804.59	−1865117.77
	21	−278.87	89166.56	1.000	−339729.45	339171.71
	22	−1289079.22^*∗*^	103009.31	0.000	−1681878.12	−896280.32
	23	−2651549.41^*∗*^	121452.39	0.000	−3116665.54	−2186433.28
	24	−227228.92	90512.62	0.719	−571803.55	117345.72
	25	−750702.70^*∗*^	99153.01	0.000	−1128519.54	−372885.86
	26	−1995687.03^*∗*^	115831.11	0.000	−2438680.97	−1552693.09
	27	221016.86	87870.91	0.716	−113519.20	555552.92

4	1	279970.62	115257.21	0.773	−158815.57	718756.81
	2	−1054590.53^*∗*^	131954.79	0.000	−1557588.23	−551592.83
	3	1315465.42^*∗*^	104377.54	0.000	917334.93	1713595.91
	5	−1397642.66^*∗*^	134765.63	0.000	−1911576.98	−883708.34
	6	1083549.13^*∗*^	105603.62	0.000	680908.27	1486189.98
	7	552301.04^*∗*^	113478.42	0.001	120246.68	984355.39
	8	−721480.51^*∗*^	129260.84	0.000	−1214023.15	−228937.87
	9	1541422.65^*∗*^	103203.59	0.000	1147589.85	1935255.45
	10	274787.23	116291.52	0.814	−167925.62	717500.08
	11	−1087566.32^*∗*^	133881.19	0.000	−1598056.55	−577076.09
	12	1326546.97^*∗*^	104917.97	0.000	926431.06	1726662.89
	13	−12059.63	118265.56	1.000	−462289.77	438170.51
	14	−1440748.24^*∗*^	136890.67	0.000	−1962967.51	−918528.97
	15	1089926.35^*∗*^	106211.88	0.000	685040.03	1494812.67
	16	553549.90^*∗*^	114403.51	0.001	117997.97	989101.82
	17	−744971.08^*∗*^	131001.24	0.000	−1244264.94	−245677.22
	18	1556899.86^*∗*^	103680.70	0.000	1161322.99	1952476.73
	19	299175.50	114154.28	0.639	−135433.36	733784.36
	20	−1003495.76^*∗*^	129940.65	0.000	−1498674.02	−508317.50
	21	1315186.55^*∗*^	103787.14	0.000	919220.09	1711153.01
	22	26386.20	115896.56	1.000	−414826.18	467598.58
	23	−1336083.99^*∗*^	132558.58	0.000	−1841428.67	−830739.31
	24	1088236.50^*∗*^	104945.84	0.000	688018.11	1488454.90
	25	564762.72^*∗*^	112482.95	0.001	136463.31	993062.13
	26	−680221.61^*∗*^	127428.18	0.000	−1165668.65	−194774.57
	27	1536482.28^*∗*^	102676.15	0.000	1144573.36	1928391.20

5	1	1677613.28^*∗*^	133154.93	0.000	1169588.98	2185637.58
	2	343052.13	147844.77	0.838	−219797.47	905901.73
	3	2713108.08^*∗*^	123857.50	0.000	2238513.33	3187702.83
	4	1397642.66^*∗*^	134765.63	0.000	883708.34	1911576.98
	6	2481191.79^*∗*^	124892.49	0.000	2002942.30	2959441.27
	7	1949943.70^*∗*^	131618.24	0.000	1447528.78	2452358.61
	8	676162.15^*∗*^	145445.44	0.003	122389.78	1229934.52
	9	2939065.31^*∗*^	122869.81	0.000	2467940.20	3410190.42
	10	1672429.89^*∗*^	134051.22	0.000	1161120.62	2183739.16
	11	310076.34	149566.65	0.940	−259310.88	879463.56
	12	2724189.64^*∗*^	124313.28	0.000	2247987.79	3200391.48
	13	1385583.03^*∗*^	135767.28	0.000	867958.68	1903207.38
	14	−43105.58	152266.43	1.000	−622779.16	536568.00
	15	2487569.01^*∗*^	125407.23	0.000	2007494.93	2967643.09
	16	1951192.56^*∗*^	132416.67	0.000	1445866.80	2456518.31
	17	652671.58^*∗*^	146994.34	0.006	93043.85	1212299.31
	18	2954542.52^*∗*^	123270.83	0.000	2482010.88	3427074.16
	19	1696818.16^*∗*^	132201.39	0.000	1192278.02	2201358.30
	20	394146.90	146049.93	0.580	−161908.66	950202.46
	21	2712829.21^*∗*^	123360.37	0.000	2239983.10	3185675.32
	22	1424028.86^*∗*^	133708.72	0.000	913975.98	1934081.74
	23	61558.67	148383.92	1.000	−503335.93	626453.27
	24	2485879.17^*∗*^	124336.79	0.000	2009594.30	2962164.03
	25	1962405.38^*∗*^	130760.94	0.000	1463106.86	2461703.90
	26	717421.05^*∗*^	143819.16	0.001	169777.97	1265064.13
	27	2934124.94^*∗*^	122427.13	0.000	2464549.00	3403700.88

6	1	−803578.50^*∗*^	103540.25	0.000	−1198199.71	−408957.30
	2	−2138139.66^*∗*^	121854.12	0.000	−2604427.06	−1671852.25
	3	231916.29	91274.47	0.697	−115566.18	579398.77
	4	−1083549.12^*∗*^	105603.62	0.000	−1486189.98	−680908.27
	5	−2481191.78^*∗*^	124892.49	0.000	−2959441.27	−2002942.30
	7	−531248.09^*∗*^	101556.44	0.000	−918178.22	−144317.96
	8	−1805029.63^*∗*^	118931.59	0.000	−2259824.58	−1350234.69
	9	457873.52^*∗*^	89929.63	0.001	115482.10	800264.95
	10	−808761.89^*∗*^	104690.38	0.000	−1207850.95	−409672.84
	11	−2171115.45^*∗*^	123937.61	0.000	−2645604.20	−1696626.69
	12	242997.85	91892.00	0.621	−106828.81	592824.51
	13	−1095608.75^*∗*^	106878.91	0.000	−1503215.45	−688002.06
	14	−2524297.37^*∗*^	127182.60	0.000	−3011570.98	−2037023.75
	15	6377.22	93366.59	1.000	−349062.36	361816.81
	16	−529999.23^*∗*^	102589.09	0.000	−920930.37	−139068.09
	17	−1828520.20^*∗*^	120820.88	0.000	−2290742.88	−1366297.53
	18	473350.73^*∗*^	90476.76	0.000	128890.47	817811.00
	19	−784373.62^*∗*^	102311.08	0.000	−1174227.05	−394520.20
	20	−2087044.88^*∗*^	119670.09	0.000	−2544742.49	−1629347.28
	21	231637.42	90598.72	0.686	−113284.44	576559.29
	22	−1057162.92^*∗*^	104251.47	0.000	−1454546.26	−659779.59
	23	−2419633.12^*∗*^	122507.69	0.000	−2888492.54	−1950773.69
	24	4687.38	91923.81	1.000	−345260.14	354634.90
	25	−518786.41^*∗*^	100442.88	0.000	−901408.56	−136164.25
	26	−1763770.74^*∗*^	116937.16	0.000	−2210731.81	−1316809.66
	27	452933.16^*∗*^	89323.84	0.001	112828.38	793037.93

7	1	−272330.42	111560.80	0.766	−697044.47	152383.64
	2	−1606891.57^*∗*^	128738.71	0.000	−2098055.07	−1115728.06
	3	763164.38^*∗*^	100280.89	0.000	380973.05	1145355.72
	4	−552301.04^*∗*^	113478.42	0.001	−984355.39	−120246.68
	5	−1949943.70^*∗*^	131618.24	0.000	−2452358.61	−1447528.78
	6	531248.09^*∗*^	101556.44	0.000	144317.96	918178.22
	8	−1273781.54^*∗*^	125976.00	0.000	−1754173.15	−793389.94
	9	989121.61^*∗*^	99058.41	0.000	611451.03	1366792.20
	10	−277513.80	112629.06	0.751	−706313.83	151286.22
	11	−1639867.36^*∗*^	130712.51	0.000	−2138740.81	−1140993.90
	12	774245.94^*∗*^	100843.28	0.000	389967.92	1158523.96
	13	−564360.66^*∗*^	114666.17	0.001	−1000973.80	−127747.53
	14	−1993049.28^*∗*^	133793.28	0.000	−2503977.02	−1482121.53
	15	537625.31^*∗*^	102188.80	0.000	148338.12	926912.51
	16	1248.86	110678.59	1.000	−420097.15	422594.87
	17	−1297272.12^*∗*^	127761.16	0.000	−1784621.36	−809922.87
	18	1004598.82^*∗*^	99555.38	0.000	625093.01	1384104.64
	19	−253125.54	110420.95	0.853	−673489.03	167237.96
	20	−1555796.79^*∗*^	126673.44	0.000	−2038905.42	−1072688.17
	21	762885.51^*∗*^	99666.23	0.000	382969.87	1142801.16
	22	−525914.83^*∗*^	112221.20	0.003	−953153.90	−98675.77
	23	−1888385.03^*∗*^	129357.50	0.000	−2381964.45	−1394805.60
	24	535935.47^*∗*^	100872.27	0.000	151549.78	920321.16
	25	12461.68	108692.23	1.000	−401323.17	426246.54
	26	−1232522.65^*∗*^	124094.83	0.000	−1705594.20	−759451.09
	27	984181.25^*∗*^	98508.77	0.000	608536.13	1359826.36

8	1	1001451.13^*∗*^	127580.66	0.000	515138.61	1487763.65
	2	−333110.02	142844.91	0.832	−876925.18	210705.14
	3	2036945.93^*∗*^	117844.27	0.000	1586035.09	2487856.77
	4	721480.51^*∗*^	129260.84	0.000	228937.87	1214023.15
	5	−676162.15^*∗*^	145445.44	0.003	−1229934.52	−122389.78
	6	1805029.63^*∗*^	118931.59	0.000	1350234.69	2259824.58
	7	1273781.55^*∗*^	125976.00	0.000	793389.94	1754173.15
	9	2262903.16^*∗*^	116805.74	0.000	1815682.87	2710123.45
	10	996267.74^*∗*^	128515.83	0.000	506491.31	1486044.17
	11	−366085.81	144626.32	0.704	−916717.97	184546.35
	12	2048027.48^*∗*^	118323.21	0.000	1595408.26	2500646.71
	13	709420.88^*∗*^	130304.81	0.000	212992.48	1205849.28
	14	−719267.73^*∗*^	147416.60	0.001	−1280610.68	−157924.78
	15	1811406.86^*∗*^	119472.02	0.000	1354674.09	2268139.63
	16	1275030.41^*∗*^	126809.95	0.000	791565.35	1758495.46
	17	−23490.57	141964.52	1.000	−563943.32	516962.18
	18	2278380.37^*∗*^	117227.50	0.000	1829663.62	2727097.12
	19	1020656.01^*∗*^	126585.14	0.000	538020.26	1503291.76
	20	−282015.25	140986.43	0.959	−818737.77	254707.27
	21	2036667.06^*∗*^	117321.65	0.000	1587615.80	2485718.32
	22	747866.71^*∗*^	128158.54	0.000	259414.81	1236318.61
	23	−614603.48^*∗*^	143402.85	0.011	−1160551.85	−68655.11
	24	1809717.01^*∗*^	118347.91	0.000	1357009.56	2262424.47
	25	1286243.23^*∗*^	125080.03	0.000	809144.39	1763342.07
	26	41258.90	138674.23	1.000	−486669.26	569187.06
	27	2257962.79^*∗*^	116339.98	0.000	1812391.29	2703534.29

9	1	−1261452.03^*∗*^	101091.25	0.000	−1647040.14	−875863.92
	2	−2596013.18^*∗*^	119780.14	0.000	−3054953.38	−2137072.98
	3	−225957.23	88486.65	0.688	−562826.58	110912.12
	4	−1541422.65^*∗*^	103203.59	0.000	−1935255.45	−1147589.85
	5	−2939065.31^*∗*^	122869.81	0.000	−3410190.42	−2467940.20
	6	−457873.52^*∗*^	89929.63	0.001	−800264.95	−115482.10
	7	−989121.61^*∗*^	99058.41	0.000	−1366792.20	−611451.03
	8	−2262903.16^*∗*^	116805.74	0.000	−2710123.45	−1815682.87
	10	−1266635.42^*∗*^	102268.93	0.000	−1656818.10	−876452.74
	11	−2628988.97^*∗*^	121899.09	0.000	−3096284.75	−2161693.19
	12	−214875.68	89123.49	0.785	−554179.86	124428.51
	13	−1553482.28^*∗*^	104508.18	0.000	−1952414.91	−1154549.65
	14	−2982170.89^*∗*^	125196.93	0.000	−3462479.53	−2501862.25
	15	−451496.30^*∗*^	90643.14	0.001	−796624.75	−106367.85
	16	−987872.75^*∗*^	100116.83	0.000	−1369663.49	−606082.02
	17	−2286393.73^*∗*^	118728.86	0.000	−2741190.31	−1831597.15
	18	15477.21	87663.58	1.000	−318250.89	349205.31
	19	−1242247.15^*∗*^	99831.93	0.000	−1622928.36	−861565.94
	20	−2544918.41^*∗*^	117557.60	0.000	−2995100.11	−2094736.71
	21	−226236.10	87789.44	0.671	−560444.15	107971.95
	22	−1515036.45^*∗*^	101819.58	0.000	−1903465.43	−1126607.47
	23	−2877506.64^*∗*^	120444.98	0.000	−3339067.93	−2415945.35
	24	−453186.14^*∗*^	89156.29	0.001	−792615.82	−113756.47
	25	−976659.93^*∗*^	97916.44	0.000	−1349890.89	−603428.97
	26	−2221644.26^*∗*^	114774.37	0.000	−2660867.50	−1782421.02
	27	−4940.37	86473.16	1.000	−334137.08	324256.34

10	1	5183.39	114421.05	1.000	−430410.41	440777.19
	2	−1329377.76^*∗*^	131225.07	0.000	−1829679.99	−829075.53
	3	1040678.19^*∗*^	103453.49	0.000	646149.29	1435207.09
	4	−274787.23	116291.52	0.814	−717500.08	167925.62
	5	−1672429.89^*∗*^	134051.22	0.000	−2183739.16	−1161120.62
	6	808761.89^*∗*^	104690.38	0.000	409672.84	1207850.95
	7	277513.80	112629.06	0.751	−151286.22	706313.83
	8	−996267.74^*∗*^	128515.83	0.000	−1486044.17	−506491.31
	9	1266635.42^*∗*^	102268.93	0.000	876452.74	1656818.10
	11	−1362353.55^*∗*^	133162.03	0.000	−1870196.94	−854510.16
	12	1051759.74^*∗*^	103998.72	0.000	655223.35	1448296.14
	13	−286846.86	117450.82	0.765	−733985.12	160291.40
	14	−1715535.47^*∗*^	136187.40	0.000	−2235180.74	−1195890.20
	15	815139.12^*∗*^	105303.92	0.000	413780.25	1216497.99
	16	278762.67	113561.07	0.757	−153567.37	711092.70
	17	−1019758.31^*∗*^	130266.19	0.000	−1516332.03	−523184.59
	18	1282112.63^*∗*^	102750.38	0.000	890166.05	1674059.21
	19	24388.27	113309.98	1.000	−406990.02	455766.56
	20	−1278282.99^*∗*^	129199.56	0.000	−1770713.20	−785852.78
	21	1040399.32^*∗*^	102857.78	0.000	648058.75	1432739.89
	22	−248401.03	115065.04	0.912	−686442.81	189640.75
	23	−1610871.22^*∗*^	131832.20	0.000	−2113535.85	−1108206.59
	24	813449.27^*∗*^	104026.83	0.000	416809.28	1210089.27
	25	289975.49	111626.01	0.656	−135034.10	714985.08
	26	−955008.84^*∗*^	126672.39	0.000	−1437639.62	−472378.06
	27	1261695.05^*∗*^	101736.64	0.000	873458.33	1649931.77

11	1	1367536.94^*∗*^	132259.72	0.000	863006.12	1872067.76
	2	32975.79	147039.03	1.000	−526796.28	592747.86
	3	2403031.74^*∗*^	122894.59	0.000	1932232.69	2873830.79
	4	1087566.32^*∗*^	133881.19	0.000	577076.09	1598056.55
	5	−310076.34	149566.65	0.940	−879463.56	259310.88
	6	2171115.45^*∗*^	123937.61	0.000	1696626.69	2645604.20
	7	1639867.36^*∗*^	130712.51	0.000	1140993.90	2138740.81
	8	366085.81	144626.32	0.704	−184546.35	916717.97
	9	2628988.97^*∗*^	121899.09	0.000	2161693.19	3096284.75
	10	1362353.55^*∗*^	133162.03	0.000	854510.16	1870196.94
	12	2414113.30^*∗*^	123353.92	0.000	1941691.71	2886534.88
	13	1075506.69^*∗*^	134889.40	0.000	561296.18	1589717.20
	14	−353181.92	151484.20	0.832	−929888.35	223524.51
	15	2177492.67^*∗*^	124456.30	0.000	1701162.05	2653823.29
	16	1641116.22^*∗*^	131516.43	0.000	1139306.88	2142925.55
	17	342595.24	146183.91	0.825	−213932.97	899123.45
	18	2644466.18^*∗*^	122303.29	0.000	2175750.18	3113182.18
	19	1386741.82^*∗*^	131299.68	0.000	885724.84	1887758.80
	20	84070.56	145234.23	1.000	−468860.77	637001.89
	21	2402752.87^*∗*^	122393.54	0.000	1933719.35	2871786.39
	22	1113952.52^*∗*^	132817.24	0.000	607376.04	1620529.00
	23	−248517.67	147581.11	0.995	−810348.55	313313.21
	24	2175802.83^*∗*^	123377.62	0.000	1703297.43	2648308.22
	25	1652329.04^*∗*^	129849.23	0.000	1156599.11	2148058.97
	26	407344.71	142990.73	0.467	−137114.52	951803.94
	27	2624048.60^*∗*^	121452.87	0.000	2158317.14	3089780.06

12	1	−1046576.35^*∗*^	102840.85	0.000	−1438609.19	−654543.52
	2	−2381137.50^*∗*^	121260.39	0.000	−2845313.84	−1916961.17
	3	−11081.55	90480.31	1.000	−355533.00	333369.89
	4	−1326546.97^*∗*^	104917.97	0.000	−1726662.89	−926431.06
	5	−2724189.63^*∗*^	124313.28	0.000	−3200391.48	−2247987.79
	6	−242997.85	91892.00	0.621	−592824.51	106828.81
	7	−774245.94^*∗*^	100843.28	0.000	−1158523.96	−389967.92
	8	−2048027.48^*∗*^	118323.21	0.000	−2500646.71	−1595408.26
	9	214875.68	89123.49	0.785	−124428.51	554179.86
	10	−1051759.74^*∗*^	103998.72	0.000	−1448296.14	−655223.35
	11	−2414113.29^*∗*^	123353.92	0.000	−2886534.88	−1941691.71
	13	−1338606.60^*∗*^	106201.50	0.000	−1743726.17	−933487.04
	14	−2767295.21^*∗*^	126613.86	0.000	−3252566.60	−2282023.83
	15	−236620.62	92590.38	0.687	−589113.40	115872.15
	16	−772997.08^*∗*^	101883.16	0.000	−1161309.63	−384684.53
	17	−2071518.05^*∗*^	120222.05	0.000	−2531607.23	−1611428.88
	18	230352.89	89675.54	0.677	−111043.20	571748.97
	19	−1027371.47^*∗*^	101603.22	0.000	−1414597.36	−640145.59
	20	−2330042.73^*∗*^	119065.48	0.000	−2785581.31	−1874504.16
	21	−11360.42	89798.59	1.000	−353223.22	330502.37
	22	−1300160.77^*∗*^	103556.88	0.000	−1694977.92	−905343.63
	23	−2662630.96^*∗*^	121917.15	0.000	−3129393.29	−2195868.64
	24	−238310.47	91135.31	0.643	−585253.51	108632.57
	25	−761784.25^*∗*^	99721.76	0.000	−1141717.29	−381851.22
	26	−2006768.58^*∗*^	116318.34	0.000	−2451507.59	−1562029.58
	27	209935.31	88512.18	0.809	−127056.37	546926.98

13	1	292030.25	116426.81	0.720	−151229.67	735290.17
	2	−1042530.90^*∗*^	132977.61	0.000	−1549316.32	−535745.48
	3	1327525.05^*∗*^	105667.63	0.000	924360.84	1730689.26
	4	12059.63	118265.56	1.000	−438170.51	462289.77
	5	−1385583.03^*∗*^	135767.28	0.000	−1903207.38	−867958.68
	6	1095608.75^*∗*^	106878.91	0.000	688002.06	1503215.45
	7	564360.67^*∗*^	114666.17	0.001	127747.53	1000973.80
	8	−709420.88^*∗*^	130304.81	0.000	−1205849.28	−212992.48
	9	1553482.28^*∗*^	104508.18	0.000	1154549.65	1952414.91
	10	286846.86	117450.82	0.765	−160291.40	733985.12
	11	−1075506.69^*∗*^	134889.40	0.000	−1589717.20	−561296.18
	12	1338606.60^*∗*^	106201.50	0.000	933487.04	1743726.17
	14	−1428688.61^*∗*^	137876.87	0.000	−1954527.02	−902850.20
	15	1101985.98^*∗*^	107479.95	0.000	692167.06	1511804.90
	16	565609.53^*∗*^	115581.75	0.001	125543.36	1005675.69
	17	−732911.45^*∗*^	132031.45	0.000	−1236027.23	−229795.67
	18	1568959.49^*∗*^	104979.35	0.000	1168309.79	1969609.19
	19	311235.13	115335.06	0.580	−127899.90	750370.16
	20	−991436.13^*∗*^	130979.20	0.000	−1490475.00	−492397.26
	21	1327246.18^*∗*^	105084.48	0.000	926212.89	1728279.47
	22	38445.83	117059.77	1.000	−407210.31	484101.97
	23	−1324024.36^*∗*^	133576.77	0.000	−1833135.39	−814913.33
	24	1100296.13^*∗*^	106229.03	0.000	695075.63	1505516.64
	25	576822.35^*∗*^	113681.10	0.001	143915.46	1009729.24
	26	−668161.98^*∗*^	128487.04	0.000	−1157564.06	−178759.90
	27	1548541.91^*∗*^	103987.35	0.000	1151503.10	1945580.72

14	1	1720718.86^*∗*^	135305.26	0.000	1204293.95	2237143.77
	2	386157.71	149784.37	0.670	−184114.52	956429.94
	3	2756213.66^*∗*^	126166.40	0.000	2272513.50	3239913.82
	4	1440748.24^*∗*^	136890.67	0.000	918528.97	1962967.51
	5	43105.58	152266.43	1.000	−536568.00	622779.16
	6	2524297.37^*∗*^	127182.60	0.000	2037023.75	3011570.98
	7	1993049.28^*∗*^	133793.28	0.000	1482121.53	2503977.02
	8	719267.73^*∗*^	147416.60	0.001	157924.78	1280610.68
	9	2982170.89^*∗*^	125196.93	0.000	2501862.25	3462479.53
	10	1715535.47^*∗*^	136187.40	0.000	1195890.20	2235180.74
	11	353181.92	151484.20	0.832	−223524.51	929888.35
	12	2767295.22^*∗*^	126613.86	0.000	2282023.83	3252566.60
	13	1428688.61^*∗*^	137876.87	0.000	902850.20	1954527.02
	15	2530674.59^*∗*^	127688.11	0.000	2041616.58	3019732.60
	16	1994298.14^*∗*^	134578.80	0.000	1480518.08	2508078.19
	17	695777.16^*∗*^	148945.01	0.003	128674.88	1262879.44
	18	2997648.10^*∗*^	125590.51	0.000	2515964.71	3479331.49
	19	1739923.74^*∗*^	134366.99	0.000	1226913.57	2252933.91
	20	437252.48	148013.05	0.391	−126335.96	1000840.92
	21	2755934.79^*∗*^	125678.40	0.000	2273944.01	3237925.57
	22	1467134.44^*∗*^	135850.29	0.000	948720.95	1985547.93
	23	104664.25	150316.55	1.000	−467620.35	676948.85
	24	2528984.75^*∗*^	126636.95	0.000	2043632.18	3014337.31
	25	2005510.96^*∗*^	132950.00	0.000	1497636.22	2513385.70
	26	760526.63^*∗*^	145812.31	0.000	205210.10	1315843.16
	27	2977230.52^*∗*^	124762.50	0.000	2498435.87	3456025.17

15	1	−809955.73^*∗*^	104160.56	0.000	−1206878.10	−413033.36
	2	−2144516.88^*∗*^	122381.64	0.000	−2612684.96	−1676348.80
	3	225539.07	91977.54	0.758	−124631.52	575709.66
	4	−1089926.35^*∗*^	106211.88	0.000	−1494812.67	−685040.03
	5	−2487569.01^*∗*^	125407.23	0.000	−2967643.09	−2007494.93
	6	−6377.22	93366.59	1.000	−361816.81	349062.36
	7	−537625.31^*∗*^	102188.80	0.000	−926912.51	−148338.12
	8	−1811406.86^*∗*^	119472.02	0.000	−2268139.63	−1354674.09
	9	451496.30^*∗*^	90643.14	0.001	106367.85	796624.75
	10	−815139.12^*∗*^	105303.92	0.000	−1216497.99	−413780.25
	11	−2177492.67^*∗*^	124456.30	0.000	−2653823.29	−1701162.05
	12	236620.63	92590.38	0.687	−115872.15	589113.40
	13	−1101985.98^*∗*^	107479.95	0.000	−1511804.90	−692167.06
	14	−2530674.59^*∗*^	127688.11	0.000	−3019732.60	−2041616.58
	16	−536376.45^*∗*^	103215.12	0.000	−929635.26	−143117.65
	17	−1834897.43^*∗*^	121352.89	0.000	−2299020.62	−1370774.24
	18	466973.51^*∗*^	91185.99	0.000	119796.29	814150.73
	19	−790750.85^*∗*^	102938.80	0.000	−1182939.79	−398561.91
	20	−2093422.11^*∗*^	120207.20	0.000	−2553042.79	−1633801.43
	21	225260.20	91307.00	0.749	−122374.19	572894.59
	22	−1063540.15^*∗*^	104867.58	0.000	−1463205.17	−663875.13
	23	−2426010.34^*∗*^	123032.41	0.000	−2896738.10	−1955282.58
	24	−1689.84	92621.95	1.000	−354302.36	350922.67
	25	−525163.63^*∗*^	101082.20	0.000	−910175.28	−140151.98
	26	−1770147.96^*∗*^	117486.76	0.000	−2219087.78	−1321208.14
	27	446555.93^*∗*^	90042.15	0.001	103691.64	789420.22

16	1	−273579.28	112501.66	0.772	−701864.87	154706.32
	2	−1608140.43^*∗*^	129554.87	0.000	−2102295.74	−1113985.11
	3	761915.52^*∗*^	101326.55	0.000	375662.95	1148168.10
	4	−553549.90^*∗*^	114403.51	0.001	−989101.82	−117997.97
	5	−1951192.56^*∗*^	132416.67	0.000	−2456518.31	−1445866.80
	6	529999.23^*∗*^	102589.09	0.000	139068.09	920930.37
	7	−1248.86	110678.59	1.000	−422594.87	420097.15
	8	−1275030.41^*∗*^	126809.95	0.000	−1758495.46	−791565.35
	9	987872.75^*∗*^	100116.83	0.000	606082.02	1369663.49
	10	−278762.67	113561.07	0.757	−711092.70	153567.37
	11	−1641116.22^*∗*^	131516.43	0.000	−2142925.55	−1139306.88
	12	772997.08^*∗*^	101883.16	0.000	384684.53	1161309.63
	13	−565609.53^*∗*^	115581.75	0.001	−1005675.69	−125543.36
	14	−1994298.14^*∗*^	134578.80	0.000	−2508078.19	−1480518.08
	15	536376.45^*∗*^	103215.12	0.000	143117.65	929635.26
	17	−1298520.98^*∗*^	128583.53	0.000	−1788890.47	−808151.48
	18	1003349.96^*∗*^	100608.57	0.000	619748.09	1386951.84
	19	−254374.40	111371.44	0.857	−678354.62	169605.83
	20	−1557045.66^*∗*^	127502.83	0.000	−2043206.74	−1070884.57
	21	761636.65^*∗*^	100718.26	0.000	377630.29	1145643.02
	22	−527163.70^*∗*^	113156.57	0.003	−957948.54	−96378.85
	23	−1889633.89^*∗*^	130169.79	0.000	−2386187.38	−1393080.39
	24	534686.61^*∗*^	101911.86	0.000	146267.76	923105.46
	25	11212.82	109657.70	1.000	−406259.09	428684.74
	26	−1233771.51^*∗*^	124941.34	0.000	−1709974.42	−757568.59
	27	982932.38^*∗*^	99573.03	0.000	603140.26	1362724.51

17	1	1024941.70^*∗*^	129343.67	0.000	531773.43	1518109.97
	2	−309619.45	144421.70	0.918	−859420.80	240181.90
	3	2060436.50^*∗*^	119750.71	0.000	1602022.29	2518850.71
	4	744971.08^*∗*^	131001.24	0.000	245677.22	1244264.94
	5	−652671.58^*∗*^	146994.34	0.006	−1212299.31	−93043.85
	6	1828520.21^*∗*^	120820.88	0.000	1366297.53	2290742.88
	7	1297272.12^*∗*^	127761.16	0.000	809922.87	1784621.36
	8	23490.57	141964.52	1.000	−516962.18	563943.32
	9	2286393.73^*∗*^	118728.86	0.000	1831597.15	2741190.31
	10	1019758.31^*∗*^	130266.19	0.000	523184.59	1516332.03
	11	−342595.24	146183.91	0.825	−899123.45	213932.97
	12	2071518.05^*∗*^	120222.05	0.000	1611428.88	2531607.23
	13	732911.45^*∗*^	132031.45	0.000	229795.67	1236027.23
	14	−695777.16^*∗*^	148945.01	0.003	−1262879.44	−128674.88
	15	1834897.43^*∗*^	121352.89	0.000	1370774.24	2299020.62
	16	1298520.98^*∗*^	128583.53	0.000	808151.48	1788890.47
	18	2301870.94^*∗*^	119143.81	0.000	1845607.60	2758134.28
	19	1044146.58^*∗*^	128361.83	0.000	554592.10	1533701.06
	20	−258524.68	142583.78	0.987	−801329.84	284280.48
	21	2060157.63^*∗*^	119236.45	0.000	1603566.40	2516748.86
	22	771357.28^*∗*^	129913.71	0.000	276085.83	1266628.73
	23	−591112.91^*∗*^	144973.57	0.022	−1143019.00	−39206.82
	24	1833207.59^*∗*^	120246.37	0.000	1373031.90	2293383.27
	25	1309733.80^*∗*^	126877.79	0.000	825619.57	1793848.03
	26	64749.47	140297.90	1.000	−469384.88	598883.82
	27	2281453.36^*∗*^	118270.67	0.000	1828272.66	2734634.06

18	1	−1276929.24^*∗*^	101578.27	0.000	−1664306.82	−889551.66
	2	−2611490.39^*∗*^	120191.47	0.000	−3071881.59	−2151099.19
	3	−241434.44	89042.64	0.570	−580414.21	97545.33
	4	−1556899.86^*∗*^	103680.70	0.000	−1952476.73	−1161322.99
	5	−2954542.52^*∗*^	123270.83	0.000	−3427074.16	−2482010.88
	6	−473350.73^*∗*^	90476.76	0.000	−817811.00	−128890.47
	7	−1004598.82^*∗*^	99555.38	0.000	−1384104.64	−625093.01
	8	−2278380.37^*∗*^	117227.50	0.000	−2727097.12	−1829663.62
	9	−15477.21	87663.58	1.000	−349205.31	318250.89
	10	−1282112.63^*∗*^	102750.38	0.000	−1674059.21	−890166.05
	11	−2644466.18^*∗*^	122303.29	0.000	−3113182.18	−2175750.18
	12	−230352.89	89675.54	0.677	−571748.97	111043.20
	13	−1568959.49^*∗*^	104979.35	0.000	−1969609.19	−1168309.79
	14	−2997648.10^*∗*^	125590.51	0.000	−3479331.49	−2515964.71
	15	−466973.51^*∗*^	91185.99	0.000	−814150.73	−119796.29
	16	−1003349.96^*∗*^	100608.57	0.000	−1386951.84	−619748.09
	17	−2301870.94^*∗*^	119143.81	0.000	−2758134.28	−1845607.60
	19	−1257724.36^*∗*^	100325.07	0.000	−1640223.15	−875225.57
	20	−2560395.62^*∗*^	117976.67	0.000	−3012062.03	−2108729.21
	21	−241713.31	88349.83	0.552	−578052.38	94625.76
	22	−1530513.66^*∗*^	102303.14	0.000	−1920716.22	−1140311.10
	23	−2892983.85^*∗*^	120854.04	0.000	−3355986.35	−2429981.35
	24	−468663.35^*∗*^	89708.14	0.000	−810183.99	−127142.72
	25	−992137.14^*∗*^	98419.18	0.000	−1367229.98	−617044.30
	26	−2237121.47^*∗*^	115203.57	0.000	−2677873.81	−1796369.13
	27	−20417.58	87042.02	1.000	−351785.08	310949.92

19	1	−19204.88	112248.20	1.000	−446527.60	408117.84
	2	−1353766.03^*∗*^	129334.84	0.000	−1847113.97	−860418.09
	3	1016289.92^*∗*^	101045.06	0.000	631131.15	1401448.69
	4	−299175.50	114154.28	0.639	−733784.36	135433.36
	5	−1696818.16^*∗*^	132201.39	0.000	−2201358.30	−1192278.02
	6	784373.63^*∗*^	102311.08	0.000	394520.20	1174227.05
	7	253125.54	110420.95	0.853	−167237.96	673489.03
	8	−1020656.01^*∗*^	126585.14	0.000	−1503291.76	−538020.26
	9	1242247.15^*∗*^	99831.93	0.000	861565.94	1622928.36
	10	−24388.27	113309.98	1.000	−455766.56	406990.02
	11	−1386741.82^*∗*^	131299.68	0.000	−1887758.80	−885724.84
	12	1027371.47^*∗*^	101603.22	0.000	640145.59	1414597.36
	13	−311235.13	115335.06	0.580	−750370.16	127899.90
	14	−1739923.74^*∗*^	134366.99	0.000	−2252933.91	−1226913.57
	15	790750.85^*∗*^	102938.80	0.000	398561.91	1182939.79
	16	254374.40	111371.44	0.857	−169605.83	678354.62
	17	−1044146.58^*∗*^	128361.83	0.000	−1533701.06	−554592.10
	18	1257724.36^*∗*^	100325.07	0.000	875225.57	1640223.15
	20	−1302671.26^*∗*^	127279.24	0.000	−1788008.68	−817333.84
	21	1016011.05^*∗*^	100435.07	0.000	633106.35	1398915.75
	22	−272789.30	112904.58	0.782	−702618.17	157039.57
	23	−1635259.49^*∗*^	129950.80	0.000	−2131010.38	−1139508.60
	24	789061.00^*∗*^	101631.99	0.000	401728.46	1176393.55
	25	265587.22	109397.65	0.774	−150890.90	682065.34
	26	−979397.11^*∗*^	124713.17	0.000	−1454755.17	−504039.05
	27	1237306.78^*∗*^	99286.58	0.000	858631.34	1615982.22

20	1	1283466.38^*∗*^	128269.37	0.000	794477.03	1772455.73
	2	−51094.77	143460.36	1.000	−597244.51	495054.97
	3	2318961.18^*∗*^	118589.54	0.000	1865117.77	2772804.59
	4	1003495.76^*∗*^	129940.65	0.000	508317.50	1498674.02
	5	−394146.90	146049.93	0.580	−950202.46	161908.66
	6	2087044.88^*∗*^	119670.09	0.000	1629347.28	2544742.49
	7	1555796.80^*∗*^	126673.44	0.000	1072688.17	2038905.42
	8	282015.25	140986.43	0.959	−254707.27	818737.77
	9	2544918.41^*∗*^	117557.60	0.000	2094736.71	2995100.11
	10	1278282.99^*∗*^	129199.56	0.000	785852.78	1770713.20
	11	−84070.56	145234.23	1.000	−637001.89	468860.77
	12	2330042.73^*∗*^	119065.48	0.000	1874504.16	2785581.31
	13	991436.13^*∗*^	130979.20	0.000	492397.26	1490475.00
	14	−437252.48	148013.05	0.391	−1000840.92	126335.96
	15	2093422.11^*∗*^	120207.20	0.000	1633801.43	2553042.79
	16	1557045.66^*∗*^	127502.83	0.000	1070884.57	2043206.74
	17	258524.68	142583.78	0.987	−284280.48	801329.84
	18	2560395.62^*∗*^	117976.67	0.000	2108729.21	3012062.03
	19	1302671.26^*∗*^	127279.24	0.000	817333.84	1788008.68
	21	2318682.31^*∗*^	118070.22	0.000	1866684.01	2770680.61
	22	1029881.96^*∗*^	128844.16	0.000	538767.50	1520996.42
	23	−332588.23	144015.92	0.844	−880860.00	215683.54
	24	2091732.26^*∗*^	119090.03	0.000	1636106.14	2547358.39
	25	1568258.48^*∗*^	125782.43	0.000	1088419.84	2048097.12
	26	323274.15	139308.10	0.838	−207075.07	853623.37
	27	2539978.04^*∗*^	117094.83	0.000	2091432.11	2988523.97

21	1	−1035215.93^*∗*^	101686.92	0.000	−1422993.18	−647438.68
	2	−2369777.08^*∗*^	120283.30	0.000	−2830492.66	−1909061.50
	3	278.87	89166.56	1.000	−339171.71	339729.45
	4	−1315186.55^*∗*^	103787.14	0.000	−1711153.01	−919220.09
	5	−2712829.21^*∗*^	123360.37	0.000	−3185675.32	−2239983.10
	6	−231637.42	90598.72	0.686	−576559.29	113284.44
	7	−762885.51^*∗*^	99666.23	0.000	−1142801.16	−382969.87
	8	−2036667.06^*∗*^	117321.65	0.000	−2485718.32	−1587615.80
	9	226236.10	87789.44	0.671	−107971.95	560444.15
	10	−1040399.32^*∗*^	102857.78	0.000	−1432739.89	−648058.75
	11	−2402752.87^*∗*^	122393.54	0.000	−2871786.39	−1933719.35
	12	11360.42	89798.59	1.000	−330502.37	353223.22
	13	−1327246.18^*∗*^	105084.48	0.000	−1728279.47	−926212.89
	14	−2755934.79^*∗*^	125678.40	0.000	−3237925.57	−2273944.01
	15	−225260.20	91307.00	0.749	−572894.59	122374.19
	16	−761636.65^*∗*^	100718.26	0.000	−1145643.02	−377630.29
	17	−2060157.63^*∗*^	119236.45	0.000	−2516748.86	−1603566.40
	18	241713.31	88349.83	0.552	−94625.76	578052.38
	19	−1016011.05^*∗*^	100435.07	0.000	−1398915.75	−633106.35
	20	−2318682.31^*∗*^	118070.22	0.000	−2770680.61	−1866684.01
	22	−1288800.35^*∗*^	102411.02	0.000	−1679399.05	−898201.65
	23	−2651270.54^*∗*^	120945.37	0.000	−3114595.23	−2187945.85
	24	−226950.04	89831.14	0.708	−568937.18	115037.09
	25	−750423.83^*∗*^	98531.31	0.000	−1125932.41	−374915.25
	26	−1995408.16^*∗*^	115299.37	0.000	−2436502.28	−1554314.04
	27	221295.73	87168.78	0.699	−110555.93	553147.39

22	1	253584.42	114019.61	0.885	−180478.50	687647.34
	2	−1080976.73^*∗*^	130875.18	0.000	−1579988.60	−581964.86
	3	1289079.22^*∗*^	103009.31	0.000	896280.32	1681878.12
	4	−26386.20	115896.56	1.000	−467598.58	414826.18
	5	−1424028.86	133708.72	0.000	−1934081.74	−913975.98
	6	1057162.92^*∗*^	104251.47	0.000	659779.59	1454546.26
	7	525914.84^*∗*^	112221.20	0.003	98675.77	953153.90
	8	−747866.71^*∗*^	128158.54	0.000	−1236318.61	−259414.81
	9	1515036.45^*∗*^	101819.58	0.000	1126607.47	1903465.43
	10	248401.03	115065.04	0.912	−189640.75	686442.81
	11	−1113952.52^*∗*^	132817.24	0.000	−1620529.00	−607376.04
	12	1300160.77^*∗*^	103556.88	0.000	905343.63	1694977.92
	13	−38445.83	117059.77	1.000	−484101.97	407210.31
	14	−1467134.44^*∗*^	135850.29	0.000	−1985547.93	−948720.95
	15	1063540.15^*∗*^	104867.58	0.000	663875.13	1463205.17
	16	527163.70^*∗*^	113156.57	0.003	96378.85	957948.54
	17	−771357.28^*∗*^	129913.71	0.000	−1266628.73	−276085.83
	18	1530513.66^*∗*^	102303.14	0.000	1140311.10	1920716.22
	19	272789.30	112904.58	0.782	−157039.57	702618.17
	20	−1029881.96^*∗*^	128844.16	0.000	−1520996.42	−538767.50
	21	1288800.35^*∗*^	102411.02	0.000	898201.65	1679399.05
	23	−1362470.19^*∗*^	131483.93	0.000	−1863851.89	−861088.49
	24	1061850.30^*∗*^	103585.11	0.000	666929.00	1456771.61
	25	538376.52^*∗*^	111214.48	0.001	114945.13	961807.91
	26	−706607.81^*∗*^	126309.88	0.000	−1187889.89	−225325.73
	27	1510096.08^*∗*^	101284.93	0.000	1123623.84	1896568.32

23	1	1616054.61^*∗*^	130920.72	0.000	1116744.62	2115364.60
	2	281493.46	145835.79	0.972	−273689.80	836676.72
	3	2651549.41^*∗*^	121452.39	0.000	2186433.28	3116665.54
	4	1336083.99^*∗*^	132558.58	0.000	830739.31	1841428.67
	5	−61558.67	148383.92	1.000	−626453.27	503335.93
	6	2419633.12^*∗*^	122507.69	0.000	1950773.69	2888492.54
	7	1888385.03^*∗*^	129357.50	0.000	1394805.60	2381964.45
	8	614603.48^*∗*^	143402.85	0.011	68655.11	1160551.85
	9	2877506.64^*∗*^	120444.98	0.000	2415945.35	3339067.93
	10	1610871.22^*∗*^	131832.20	0.000	1108206.59	2113535.85
	11	248517.67	147581.11	0.995	−313313.21	810348.55
	12	2662630.97^*∗*^	121917.15	0.000	2195868.64	3129393.29
	13	1324024.36^*∗*^	133576.77	0.000	814913.33	1833135.39
	14	−104664.25	150316.55	1.000	−676948.85	467620.35
	15	2426010.34^*∗*^	123032.41	0.000	1955282.58	2896738.10
	16	1889633.89^*∗*^	130169.79	0.000	1393080.39	2386187.38
	17	591112.91^*∗*^	144973.57	0.022	39206.82	1143019.00
	18	2892983.85^*∗*^	120854.04	0.000	2429981.35	3355986.35
	19	1635259.49^*∗*^	129950.80	0.000	1139508.60	2131010.38
	20	332588.23	144015.92	0.844	−215683.54	880860.00
	21	2651270.54^*∗*^	120945.37	0.000	2187945.85	3114595.23
	22	1362470.19^*∗*^	131483.93	0.000	861088.49	1863851.89
	24	2424320.50^*∗*^	121941.13	0.000	1957473.14	2891167.85
	25	1900846.71^*∗*^	128485.12	0.000	1410452.21	2391241.21
	26	655862.38^*∗*^	141753.14	0.003	116153.09	1195571.67
	27	2872566.27^*∗*^	119993.34	0.000	2412592.55	3332539.99

24	1	−808265.88^*∗*^	102869.27	0.000	−1200403.79	−416127.98
	2	−2142827.03^*∗*^	121284.50	0.000	−2607088.98	−1678565.09
	3	227228.92	90512.62	0.719	−117345.72	571803.55
	4	−1088236.50^*∗*^	104945.84	0.000	−1488454.90	−688018.11
	5	−2485879.16^*∗*^	124336.79	0.000	−2962164.03	−2009594.30
	6	−4687.38	91923.81	1.000	−354634.90	345260.14
	7	−535935.47^*∗*^	100872.27	0.000	−920321.16	−151549.78
	8	−1809717.01^*∗*^	118347.91	0.000	−2262424.47	−1357009.56
	9	453186.15^*∗*^	89156.29	0.001	113756.47	792615.82
	10	−813449.27^*∗*^	104026.83	0.000	−1210089.27	−416809.28
	11	−2175802.83^*∗*^	123377.62	0.000	−2648308.22	−1703297.43
	12	238310.47	91135.31	0.643	−108632.57	585253.51
	13	−1100296.13^*∗*^	106229.03	0.000	−1505516.64	−695075.63
	14	−2528984.74^*∗*^	126636.95	0.000	−3014337.31	−2043632.18
	15	1689.84	92621.95	1.000	−350922.67	354302.36
	16	−534686.61^*∗*^	101911.86	0.000	−923105.46	−146267.76
	17	−1833207.58^*∗*^	120246.37	0.000	−2293383.27	−1373031.90
	18	468663.36^*∗*^	89708.14	0.000	127142.72	810183.99
	19	−789061.00^*∗*^	101631.99	0.000	−1176393.55	−401728.46
	20	−2091732.26^*∗*^	119090.03	0.000	−2547358.39	−1636106.14
	21	226950.04	89831.14	0.708	−115037.09	568937.18
	22	−1061850.30^*∗*^	103585.11	0.000	−1456771.61	−666929.00
	23	−2424320.49^*∗*^	121941.13	0.000	−2891167.85	−1957473.14
	25	−523473.79^*∗*^	99751.07	0.000	−903516.01	−143431.56
	26	−1768458.12^*∗*^	116343.47	0.000	−2213287.24	−1323628.99
	27	448245.78^*∗*^	88545.21	0.001	111127.54	785364.01

25	1	−284792.10	110548.05	0.672	−705670.28	136086.08
	2	−1619353.25^*∗*^	127862.10	0.000	−2107312.57	−1131393.93
	3	750702.70^*∗*^	99153.01	0.000	372885.86	1128519.54
	4	−564762.72^*∗*^	112482.95	0.001	−993062.13	−136463.31
	5	−1962405.38^*∗*^	130760.94	0.000	−2461703.90	−1463106.86
	6	518786.41^*∗*^	100442.88	0.000	136164.25	901408.56
	7	−12461.68	108692.23	1.000	−426246.54	401323.17
	8	−1286243.23^*∗*^	125080.03	0.000	−1763342.07	−809144.39
	9	976659.93^*∗*^	97916.44	0.000	603428.97	1349890.89
	10	−289975.49	111626.01	0.656	−714985.08	135034.10
	11	−1652329.04^*∗*^	129849.23	0.000	−2148058.97	−1156599.11
	12	761784.25^*∗*^	99721.76	0.000	381851.22	1141717.29
	13	−576822.35^*∗*^	113681.10	0.001	−1009729.24	−143915.46
	14	−2005510.96^*∗*^	132950.00	0.000	−2513385.70	−1497636.22
	15	525163.63^*∗*^	101082.20	0.000	140151.98	910175.28
	16	−11212.82	109657.70	1.000	−428684.74	406259.09
	17	−1309733.80^*∗*^	126877.79	0.000	−1793848.03	−825619.57
	18	992137.14^*∗*^	98419.18	0.000	617044.30	1367229.98
	19	−265587.22	109397.65	0.774	−682065.34	150890.90
	20	−1568258.48^*∗*^	125782.43	0.000	−2048097.12	−1088419.84
	21	750423.83^*∗*^	98531.31	0.000	374915.25	1125932.41
	22	−538376.52^*∗*^	111214.48	0.001	−961807.91	−114945.13
	23	−1900846.71^*∗*^	128485.12	0.000	−2391241.21	−1410452.21
	24	523473.79^*∗*^	99751.07	0.000	143431.56	903516.01
	26	−1244984.33^*∗*^	123185.18	0.000	−1714700.19	−775268.47
	27	971719.56^*∗*^	97360.36	0.000	600543.78	1342895.34

26	1	960192.23^*∗*^	125723.50	0.000	481088.89	1439295.57
	2	−374368.92	141188.68	0.616	−911914.42	163176.58
	3	1995687.03^*∗*^	115831.11	0.000	1552693.09	2438680.97
	4	680221.61^*∗*^	127428.18	0.000	194774.57	1165668.65
	5	−717421.05^*∗*^	143819.16	0.001	−1265064.13	−169777.97
	6	1763770.74^*∗*^	116937.16	0.000	1316809.66	2210731.81
	7	1232522.65^*∗*^	124094.83	0.000	759451.09	1705594.20
	8	−41258.90	138674.23	1.000	−569187.06	486669.26
	9	2221644.26^*∗*^	114774.37	0.000	1782421.02	2660867.50
	10	955008.84^*∗*^	126672.39	0.000	472378.06	1437639.62
	11	−407344.71	142990.73	0.467	−951803.94	137114.52
	12	2006768.59^*∗*^	116318.34	0.000	1562029.58	2451507.59
	13	668161.98^*∗*^	128487.04	0.000	178759.90	1157564.06
	14	−760526.63^*∗*^	145812.31	0.000	−1315843.16	−205210.10
	15	1770147.96^*∗*^	117486.76	0.000	1321208.14	2219087.78
	16	1233771.51^*∗*^	124941.34	0.000	757568.59	1709974.42
	17	−64749.47	140297.90	1.000	−598883.82	469384.88
	18	2237121.47^*∗*^	115203.57	0.000	1796369.13	2677873.81
	19	979397.11^*∗*^	124713.17	0.000	504039.05	1454755.17
	20	−323274.15	139308.10	0.838	−853623.37	207075.07
	21	1995408.16^*∗*^	115299.37	0.000	1554314.04	2436502.28
	22	706607.81^*∗*^	126309.88	0.000	225325.73	1187889.89
	23	−655862.38^*∗*^	141753.14	0.003	−1195571.67	−116153.09
	24	1768458.12^*∗*^	116343.47	0.000	1323628.99	2213287.24
	25	1244984.33^*∗*^	123185.18	0.000	775268.47	1714700.19
	27	2216703.89^*∗*^	114300.33	0.000	1779165.65	2654242.13

27	1	−1256511.66^*∗*^	100552.72	0.000	−1640125.31	−872898.01
	2	−2591072.81^*∗*^	119325.99	0.000	−3048414.59	−2133731.03
	3	−221016.86	87870.91	0.716	−555552.92	113519.20
	4	−1536482.28^*∗*^	102676.15	0.000	−1928391.20	−1144573.36
	5	−2934124.94^*∗*^	122427.13	0.000	−3403700.88	−2464549.00
	6	−452933.16^*∗*^	89323.84	0.001	−793037.93	−112828.38
	7	−984181.25^*∗*^	98508.77	0.000	−1359826.36	−608536.13
	8	−2257962.79^*∗*^	116339.98	0.000	−2703534.29	−1812391.29
	9	4940.37	86473.16	1.000	−324256.34	334137.08
	10	−1261695.05^*∗*^	101736.64	0.000	−1649931.77	−873458.33
	11	−2624048.60^*∗*^	121452.87	0.000	−3089780.06	−2158317.14
	12	−209935.31	88512.18	0.809	−546926.98	127056.37
	13	−1548541.91^*∗*^	103987.35	0.000	−1945580.72	−1151503.10
	14	−2977230.52^*∗*^	124762.50	0.000	−3456025.17	−2498435.87
	15	−446555.93^*∗*^	90042.15	0.001	−789420.22	−103691.64
	16	−982932.38^*∗*^	99573.03	0.000	−1362724.51	−603140.26
	17	−2281453.36^*∗*^	118270.67	0.000	−2734634.06	−1828272.66
	18	20417.58	87042.02	1.000	−310949.92	351785.08
	19	−1237306.78^*∗*^	99286.58	0.000	−1615982.22	−858631.34
	20	−2539978.04^*∗*^	117094.83	0.000	−2988523.97	−2091432.11
	21	−221295.73	87168.78	0.699	−553147.39	110555.93
	22	−1510096.08^*∗*^	101284.93	0.000	−1896568.32	−1123623.84
	23	−2872566.27^*∗*^	119993.34	0.000	−3332539.99	−2412592.55
	24	−448245.78^*∗*^	88545.21	0.001	−785364.01	−111127.54
	25	−971719.56^*∗*^	97360.36	0.000	−1342895.34	−600543.78
	26	−2216703.89^*∗*^	114300.33	0.000	−2654242.13	−1779165.65

^*∗*^The mean difference is significant at the 0.05 level.

**Table 9:** Differences.

Policy combination																										
	2	3	4	5	6	7	8	9	10	11	12	13	14	15	16	17	18	19	20	21	22	23	24	25	26	27
Dif1	✓	✓	✓	✓	✓	×	✓	✓	×	✓	✓	×	✓	✓	✓	✓	✓	×	✓	✓	✓	✓	✓	×	✓	✓
Dif2	✓	✓	×	✓	✓	×	✓	✓	×	✓	✓	×	✓	✓	×	✓	✓	×	✓	✓	×	✓	✓	×	✓	✓
Dif3	✓	✓	×	✓	✓	×	✓	✓	×	✓	✓	×	✓	✓	×	✓	✓	×	✓	✓	×	✓	✓	×	✓	✓

*Note.* Dif1 refers to whether there is a significant difference in the economic effect with policy combination 1, Dif2 refers to whether there is a significant difference in the STI effect with policy Combination 1, and Dif3 refers to whether there is a significant difference in the economic effect and STI effect with policy Combination 1 at the same time.

## Data Availability

The data used to support the findings of this study are included within the article.

## References

[1] Zhang Y., Geng Z., Wang Y. (2015). The complex adaptation of regional science and technology innovation policy. *Chinese Journal of Systems Science*.

[2] Peng J., Zhong W., Sun W. (2008). Policy measurement, policy collaborative evolution and economic performance: an empirical study based on innovation policy. *Management World*.

[3] Surianto S., Syahirul A., Nindrea R. D., Trisnantoro L. (2019). Regional policy for disaster risk management in developing countries within the sendai framework: a systematic review. *Open Access Macedonian Journal of Medical Sciences*.

[4] Krivosheev D. S., Troitskiy E. F., Troitskiy E. F. (2019). The EU regional policy in wallonia (2000-17). *Vestnik Tomskogo gosudarstvennogo universiteta*.

[5] Shi J., Chen X., Liu Y. (2011). Research of district strategy’s promotion mechanism on Guangdong industry transfer. *Research of Finance and Accounting*.

[6] Yu S. (2014). The influence of regional economic policy on regional economic development. *China Business and Trade*.

[7] Lan X., Wang S., Zhu Z. (2017). Analysis of the impact of regional economic policies on regional economic development. *Modern Business*.

[8] Lin X. (2016). The impact of EU’s regional policy on the Portuguese national capability in terms of science, technology and innovation. *Forum on Science and Technology in China*.

[9] Wang J., Zhang Y. (2018). A study on the influence mechanism of science, technology and innovation policy on regional innovation capacity based on system dynamics. *Science and Technology Management Research*.

[10] Avdiushchenko A., Zając P. (2019). Circular economy indicators as a supporting tool for European regional development policies. *Sustainability*.

[11] O’Brien M., Burrows S. (2019). Assessing the effectiveness of regional policy responses to mass redundancies: the case of the illawarra region, Australia. *Economic Papers: A journal of applied economics and policy*.

[12] Gülal F., Ayaita A. (2019). The impact of minimum wages on well-being: evidence from a quasi-experiment in Germany. *Journal of Happiness Studies*.

[13] Cheng X, Bao X., Zhang R., Shen X. (2018). Analysis on the efficiency of science and technology financial policy in beijing, tianjin and hebei region based on policy quantification. *Science and Technology Management Research*.

[14] Deng J., Long R. (2013). Empirical research on venture capital to promote technological innovation in China. *Technoeconomics & Management Research*.

[15] Research Yu. (2013). On the vertical gap of bank loans, government and enterprise science and technology investment contribution——estimation based on quantile regression of panel data. *Science & Technology Progress and Policy*.

[16] Fan Z. (2018). The technological innovation effect, existing problems and strategic choice of intellectual property protection. *Scientific Management Research*.

[17] Wang Z., Deng S. (2017). Research on policy performance of coordinated development of intellectual property protection and science and technology innovation in Jiangxi Province. *Enterprise Economy*.

[18] Zhuang Y., Li Y., Qin Y. (2012). Innovation environment incentive and enterprise growth: an empirical study based on innovative enterprises in Central China.

[19] Álvarez-Ayuso I. C., Kao C., Romero-Jordán D. (2018). Long run effect of public grants and tax credits on R&D investment: a non-stationary panel data approach. *Economic Modelling*.

[20] Raffaello B., Paolo P. (2016). The impact of R&D subsidies on firm innovation. *Research Policy*.

[21] Mukherjee A., Singh M., Žaldokas A. (2017). Do corporate taxes hinder innovation?. *Journal of Financial Economics*.

[22] Cappelen Å, Raknerud A, Rybalka M (2012). The effects of R&D tax credits on patenting and innovations. *Research Policy*.

[23] Guerzoni M., Raiteri E. (2015). Demand-side vs. supply-side technology policies: hidden treatment and new empirical evidence on the policy mix. *Research Policy*.

[24] Aschhoff B., Sofka W. (2009). Innovation on demand-Can public procurement drive market success of innovations?. *Research Policy*.

[25] Song H., Zhang S. (2014). China’s government procurement policy system construction and development for innovation product. *Studies in Science of Science*.

[26] Hu K., Cai H., Wu Q. (2013). Has government procurement in China promoted technological innovation?. *Journal of Finance and Economics*.

[27] Kalcheva I., Mclemore P., Pant S. (2018). Innovation: the interplay between demand-side shock and supply-side environment. *Research Policy*.

[28] Dou S., Liu J., Pang S. (2019). Synergy effects of policy mix in technological innovation——based on perspective of supply side-demand side. *Science & Technology Progress and Policy*.

[29] Guo Y., Ge J., Cheng C., Duan S. (2019). Supply mode of technology policies mix based on QCA method. *Soft Science*.

[30] Qin X. (2018). *Technology Subsidies, Tax Preferences and enterprise Innovation: The Effect of R&D Policies of Listed Companies*.

[31] Romer P. M. (1986). Increasing returns and long-run growth. *Journal of Political Economy*.

[32] Fox J. (2016). *Applied Regression Analysis and Generalized Linear Models*.

[33] Kleinbaum D. G., Kupper L. L., Nizam A., Rosenberg E. S. (2008). *Applied Regression Analysis and Other Multivariable Methods*.

[34] Zhang J. (2008). The method for multiple comparisons. *Journal of Evidence-Based Medicine*.

[35] Shingala M. C., Rajyaguru D. A. (2015). Comparison of post hoc tests for unequal variance. *International Journal of New Technologies in Science and Engineering*.

[36] Games P. A., Howell J. F. (1976). Pairwise multiple comparison procedures with unequal N’s and/or variances: a Monte Carlo study. *Journal of Educational Statistics*.

[37] Rothwell R., Zegveld W. (1981). *Industrial Innovation and Public Policy: Preparing for the 1980s and the 1990s*.

